# 3D Lung-on-Chip Model Based on Biomimetically Microcurved Culture Membranes

**DOI:** 10.1021/acsbiomaterials.1c01463

**Published:** 2022-05-03

**Authors:** Danielle Baptista, Liliana Moreira Teixeira, David Barata, Zeinab Tahmasebi Birgani, Jasia King, Sander van Riet, Thijs Pasman, André A. Poot, Dimitrios Stamatialis, Robbert J. Rottier, Pieter S. Hiemstra, Aurélie Carlier, Clemens van Blitterswijk, Pamela Habibović, Stefan Giselbrecht, Roman Truckenmüller

**Affiliations:** † MERLN Institute for Technology-Inspired Regenerative Medicine, Maastricht University, Universiteitssingel 40, 6229 ER Maastricht, The Netherlands; ‡ Department of Developmental BioEngineering, Technical Medical Centre, University of Twente, Drienerlolaan 5, 7522 NB Enschede, The Netherlands; § Instituto de Medicina Molecular, Faculty of Medicine, University of Lisbon, Avenida Professor Egas Moniz, 1649-028 Lisbon, Portugal; ∥ Department of Pulmonology, Leiden University Medical Center, Albinusdreef 2, 2333 ZA Leiden, The Netherlands; ⊥ Department of Biomaterials Science and Technology, Technical Medical Centre, University of Twente, Drienerlolaan 5, 7522 NB Enschede, The Netherlands; # Department of Pediatric Surgery/Cell Biology, Erasmus (University) Medical Center Rotterdam − Sophia Children’s Hospital, Doctor Molewaterplein 40, 3015 GD Rotterdam, The Netherlands

**Keywords:** curvature, alveolar epithelial cells, biomimetics, ion track-etched membranes, microthermoforming, organ on a chip (OoC)

## Abstract

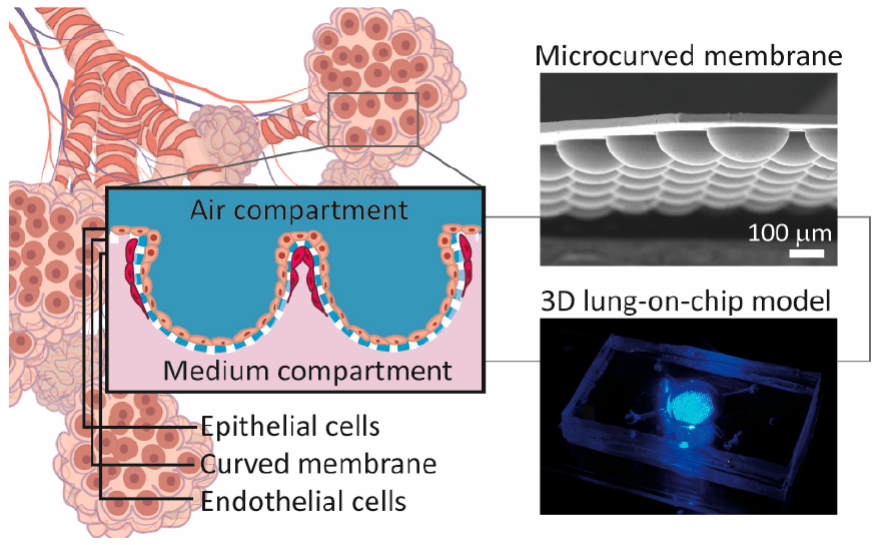

A comparatively straightforward approach to accomplish more physiological realism in organ-on-a-chip (OoC) models is through substrate geometry. There is increasing evidence that the strongly, microscale curved surfaces that epithelial or endothelial cells experience when lining small body lumens, such as the alveoli or blood vessels, impact their behavior. However, the most commonly used cell culture substrates for modeling of these human tissue barriers in OoCs, ion track-etched porous membranes, provide only flat surfaces. Here, we propose a more realistic culture environment for alveolar cells based on biomimetically microcurved track-etched membranes. They recreate the mainly spherical geometry of the cells’ native microenvironment. In this feasibility study, the membranes were given the shape of hexagonally arrayed hemispherical microwells by an innovative combination of three-dimensional (3D) microfilm (thermo)forming and ion track technology. Integrated in microfluidic chips, they separated a top from a bottom cell culture chamber. The microcurved membranes were seeded by infusion with primary human alveolar epithelial cells. Despite the pronounced topology, the cells fully lined the alveoli-like microwell structures on the membranes’ top side. The confluent curved epithelial cell monolayers could be cultured successfully at the air−liquid interface for 14 days. Similarly, the top and bottom sides of the microcurved membranes were seeded with cells from the Calu-3 lung epithelial cell line and human lung microvascular endothelial cells, respectively. Thereby, the latter lined the interalveolar septum-like interspace between the microwells in a network-type fashion, as in the natural counterpart. The coculture was maintained for 11 days. The presented 3D lung-on-a-chip model might set the stage for other (micro)anatomically inspired membrane-based OoCs in the future.

## 1 Introduction

The primary task of our lung(s) is to facilitate gas exchange between the lung lumen and the vascular space. The lung’s epithelial lining also functions as a protective physical and immunological barrier that prevents inhaled toxins and pathogens from contacting the subepithelial tissue.^1^ In adult humans, the gas exchange is mainly accomplished by around 80 m^2^ total surface area^2^ of the on average roughly 480 million pulmonary alveoli,^3^ considering both lungs together. Most of the existing knowledge and understanding of lung development, (patho)physiology, and pharmacokinetics and -dynamics stems from animal models.^4^ Besides negative ethical and also economic implications of animal testing, these models often only poorly recapitulate lung anatomy and physiology of humans. This emphasizes the relevance of *in vitro* models of the human lung^4−9^ that are predictive because they are realistic, reliable, and reproducible.

An exciting and promising development in the field of *in vitro* models are organ-on-a-chip (OoC) models.^10−12^ This includes OoC models of (tissues of) the lung.^13−17^ The desired and essential physiological realism in OoCs can be accomplished basically by two approaches: (i) correspondingly high biological complexity or (ii) artificially engineered physiologically relevant microenvironments mimicking geometrical, mechanical, or biochemical key aspects of the tissue or organ of interest.^18^ The first approach means in the first instance complex multicellular (coculture) systems. An example for such a system in the field of lung research is a biomimetic model of the airways using airway epithelial cells, lung fibroblasts, and microvascular endothelial cells.^19^ The second approach can be implemented without significantly compromising the robustness of OoCs. A prototypic example for this approach again in the lung field and potentially the most popular OoC is the human alveolar−capillary interface mimic of Huh et al.^20^ The lung on a chip includes cyclic mechanical stretching of its elastic culture membrane through lateral vacuum actuator channels. This model was followed by others with even more realistic extension/dilation of the culture substrate.^21,22^ Another microenvironmental factor that is technically comparatively straightforward to implement is, as already indicated, substrate geometry, including substrate topography/topology.

More and more studies evidence that surface curvature with curvature radii in a cell size-near range impacts cell behavior.^23−25^ Epithelial and endothelial cells lining small acinar or tubular body lumens of internal human barrier tissues, such as in the lung alveoli, kidney tubules, or small-diameter blood vessels, experience such strongly, milli- to micrometer-scale curved surfaces. The probably most commonly employed cell culture substrates for *in vitro* modeling of human tissue barriers, such as the air−blood barrier in the lungs, are ion track-etched porous membranes^26,27^ from polycarbonate (PC) or polyethylene terephthalate (PET). In these barrier model applications, the track-etched membranes are typically integrated into cell culture inserts/Boyden chambers in multiwell plates, microfluidic chips/OoC devices, or a combination of both. There, they separate an upper/top from a lower/bottom well or (perfusable) compartment. The membranes allow the establishment of cocultures or confrontation cultures (in each case with the two cell populations on either side of the porous substrate), air−liquid interface (ALI) cultures, or combinations thereof. In cell culture applications, track-etched membranes outperform most of their membrane counterparts based on other pore-forming processes concerning a number of aspects.^28^ However, so far, these membrane-based culture environments provide only flat, two-dimensional surfaces. Regarding substrate geometry, this flatness makes the membranes largely unspecific for the barrier tissue or the corresponding organ to be researched, the (micro)anatomy of which they do not adequately reflect. To more closely represent *in vitro* the spatial cell organization in conjunction with tissue- or organ-specific curvature *in vivo*, also in light of the above-mentioned influence of near-cell-scale curvature on (epithelial/endothelial) cell behavior, it would be necessary to add the feature of microcurvature to track-etched culture membranes. Such a biomimetically microcurved culture membrane is at the center of the three-dimensional (3D) lung-on-chip model presented in this paper.

**Figure 1:**
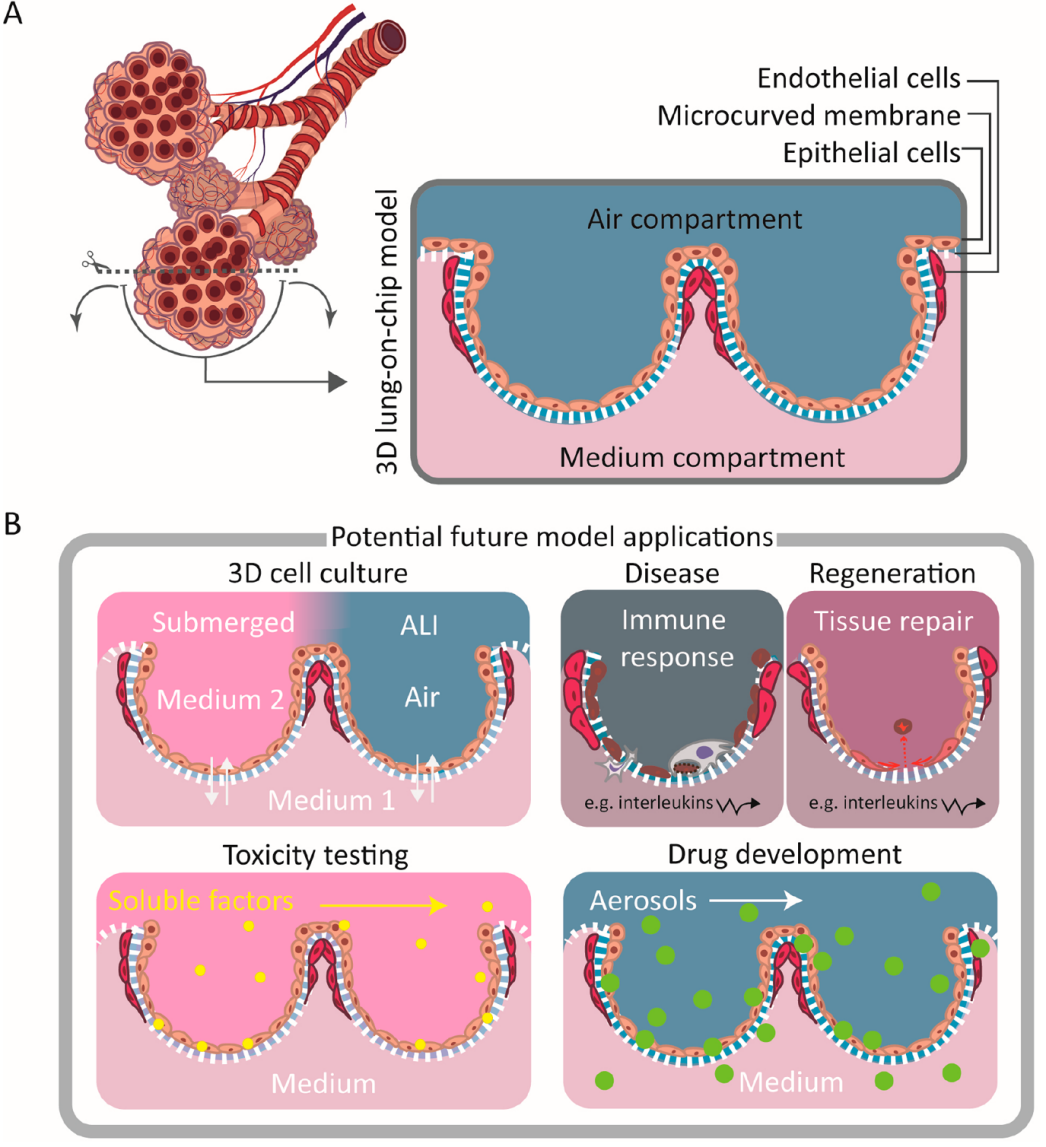
Concept of the 3D lung-on-a-chip model based on biomimetically microcurved culture membranes. (A) We approached a structure similar to a cut and flipped open alveolar sac as a cell-populated membrane with the microcurved shape, size, and also arrangement of its bioartificial alveoli mimicking the ones of the adult organ. Integrated in microfluidic chips/OoC devices where the microcurved membranes separate a top from a bottom compartment, they can be seeded by infusion with lung epithelial and microvascular endothelial cells on the top and bottom side of the membrane. The spatial cell distribution is then similar to the alveolar–capillary barrier. (B) Potential future applications of the model include 3D ALI culture (following submerged culture), modeling of disease and repair/regeneration, and toxicity and pharmaceutical efficacy testing (temporarily under submerged conditions or exposed to vapors or aerosols).

Here, we propose a more realistic environment for the culture of human lung epithelial cells in microfluidic chips, also at the ALI and in coculture with human lung microvascular endothelial cells. It is based on biomimetically microcurved porous culture membranes (Figure 1A). This feasibility study is about their microtechnological fabrication, microfluidic integration, geometrical characterization, and permeability testing, and a first cell culture demonstration on these membranes. They were given the shape of arrays of honeycomb-type, hexagonally arranged round-/U-bottom microwells with a hemispherical cross-section. The shaping of the membranes was accomplished by an innovative combination of the well-established processes (3D) microthermoforming^29,30^ (of polymer films) and ion track technology.^26^ The biomimetic membranes recreate the mainly spherical geometry of the native microenvironment of the alveolar epithelial cells, the alveolus, including the spatial configuration of multiple of these microenvironments in the alveolar sac. Additionally, the space between adjacent microwells to a bigger extent reproduces the lumen of the folded double epithelial monolayer wall between adjacent alveoli, the interalveolar septum.^31−33^ This lumen also contains (a network of) extracellular matrix-embedded blood capillaries. In contrast to typical lung-on-chip models, our 3D model does not represent the alveolar capillary barrier where, separated by a very thin basement membrane, the alveolar space is nearest to the lumen of the blood capillaries, and also not only a single alveolus. Instead, as indicated, it represents a structure similar to an alveolar sac, one that is cut and flipped open. The microfluidic lung on chip might in the not too distant future allow the controlled modeling of the delivery of pharmaceutical compounds or the exposure to toxic substances and/or (respirable) particles in temporal medium phases, vapors or aerosols, also smoke (Figure 1B). Thereby, for example, in the field of drug delivery and uptake via inhalation, the biomimetic 3D character of the model may enable such studies in a more representative spatial context.

## 2 Experimental Section

### 2.1 Rationale of Design, Microfabrication, Visual Inspection, Geometrical Characterization, and Permeability Testing of Biomimetic Culture Membranes

Microcurved porous membranes were fabricated from (flat) 25 μm thin ion track-etched PC films with a pore diameter of 0.4 μm and a pore density of 1 × 10^6^ pores cm^–2^ (it4ip) as semifinished products. Pore diameters of 0.4 and 0.8 μm and pore densities of around 1–5 × 10^6^ pores cm^–2^ are probably the most common choice for the culture of lung epithelial cells at the ALI.^34−38^ Hexagonally arranged and square-arranged arrays of hemispherically shaped microwells with a maximum inner diameter (at the transition from the microwells’ concave inner surface to their convex circular upper rim) of approximately 200 μm were formed in the porous PC films (Figure 1A). These mimic key aspects of the anatomy of human alveoli in terms of their average shape, size^3^ and, in the case of the hexagonal configuration, also arrangement.

**Figure 2:**
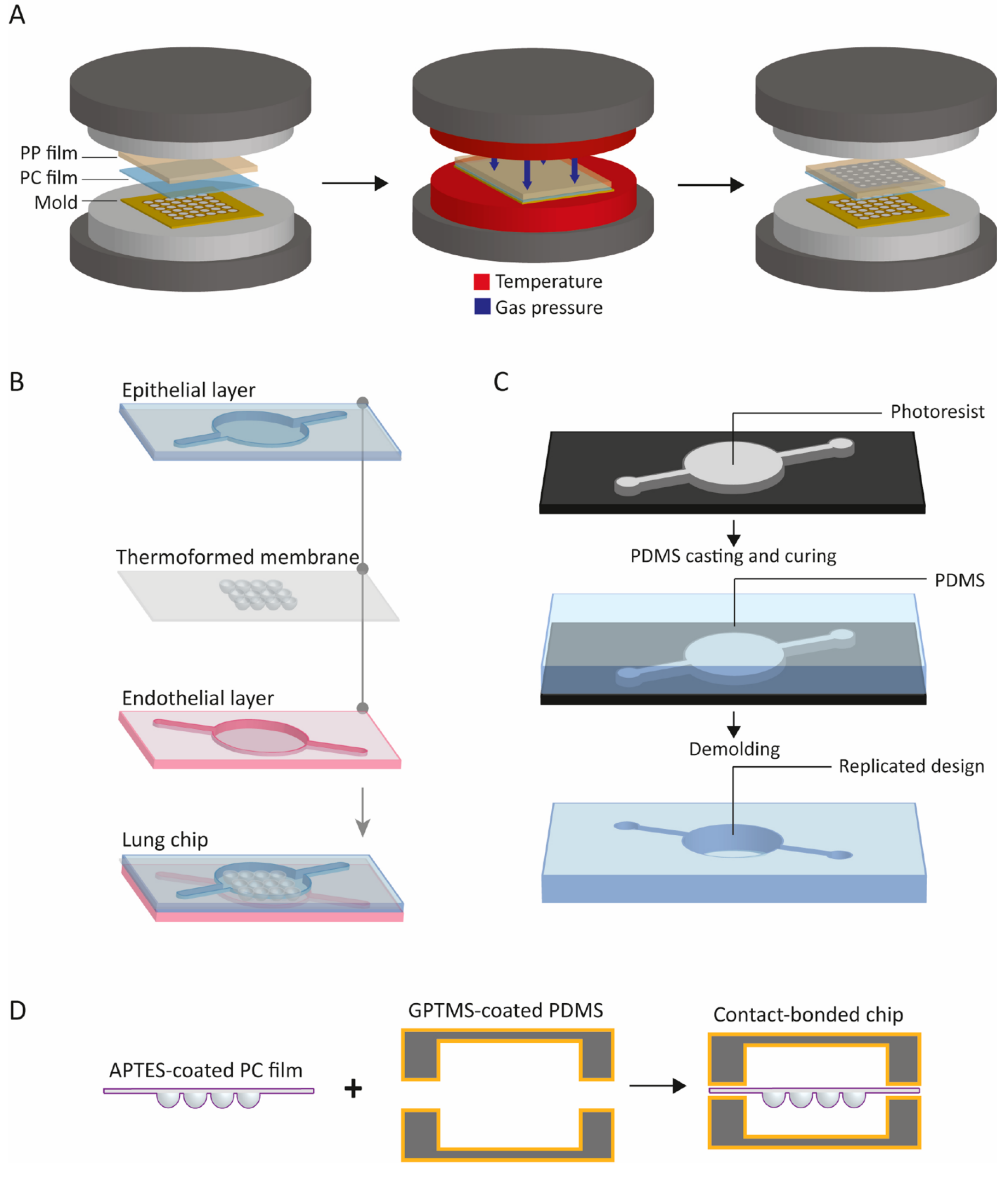
Microfabrication of the 3D lung-on-chip device. (A) Fabrication of the biomimetically microcurved culture membrane by thermoforming. An additional (dense) PP sealing film allows the micro pressure (thermo)forming of the porous PC film/membrane using compressed nitrogen. (B) Microfluidic chip construction and design. The OoC device consists of a top and a bottom housing half from polydimethylsiloxane (PDMS) containing microfluidic structures and the biomimetic membrane sandwiched in between. (C) Fabrication of the housing halves of the PDMS chip body by casting of the PDMS precursor over an SU-8-silicon mold, curing of the precursor, and peeling off the structured PDMS layer. (D) Assembly and chemical−thermal bonding of the housing halves and the microcurved membrane.

For forming, a novel variant of a previously described free-forming version of the micro pressure (thermo)forming technique^28,30,39^ was employed (Figure 2A). In free forming,^40^ the film or sheet material, which is laterally fixated at a circumferential contour, takes a bubble-type shape. This is the result (of the geometry of the fixation contour and) of the equilibrium between the differential pressure applied on the semifinished product through vacuum or compressed gas to (de)form it and the material−internal tension as a consequence of the applied forming pressure. Thereby, the formed portion of the material does not touch the surface of a mold. For the micro free-forming variant, together with the film to be formed, another film was introduced and inserted in the forming process and equipment, respectively. This (dense) sealing film allowed the film to be formed to be porous by preventing the compressed nitrogen here used as forming gas to leak through the pores during forming. Concretely, in the novel process, first, the porous PC film/membrane was laminated with a (dense) 50 μm thin polypropylene (PP) film (DURABLE). Then, the stack of the two films was loaded into a self-built microthermoforming machine. This was based on a heated laboratory press. Between the two heated press platens, a generic tool with a connection to the pressurized forming gas was mounted. The tool in turn was designed to receive exchangeable sheet-type micromolds from brass with application-specific cavity designs fabricated by mechanical micromachining. In this study, the molds from 0.3 mm thin Cu(63/)Zn37 (Ms63) sheets contained arrayed microcavities in the form of circular-cylindrical through-holes. The microcavities were fabricated by microdrilling using hard metal tools on CNC precision machining equipment including a high-speed spindle (i-sys Mikro- und Feinwerktechnik). In the thermoforming machine, the film stack was inflated into the mold cavities at a forming temperature and pressure of 153 °C and 15 bar, respectively. For this purpose, after the films were heated and reached their forming temperature, the nitrogen pressure was immediately applied on the films and automatically ramped up. Then, after first having reached the forming temperature and now also reaching the forming pressure, which was the case within a few seconds, the brief constant heating of the films was instantaneously ended. So, there was factually no dwell time. When, without further heating, the temperature dropped to around 100 °C, the gas pressure on the films was released. Finally, the film stack was detached from the mold, and the formed porous film was separated from the similarly formed sealing film by peeling them from each other.

The microthermoformed membranes were geometrically characterized by the maximum inner depth of the hemispherical microwells, which can be found at their horizontal center, using a VK-X250 confocal laser scanning microscope-based profilometer in combination with the MultiFileAnalyzer image analysis software (both KEYENCE). The membranes and their details were visually inspected and geometrically characterized by the size/diameter of their micropores using a JSM-IT200 InTouchScope scanning electron microscope (SEM; JEOL) and the ImageJ-based open-source image processing software Fiji (https://fiji.sc/), respectively.

The formed membranes (with the hexagonal array design) were also tested for their permeability in comparison with the unformed membrane semifinished products. For this, the membranes were mounted in bottom-less culture inserts (CellCrown24NX, Scaffdex Oy), with polydimethylsiloxane (PDMS) as a sealant. Anionic, fluorescein isothiocyanate (FITC)-labeled dextran with a molecular weight of 3000 g mol^−1^ (3 kDa; Invitrogen) and provided in powder form was dissolved in phosphate-buffered saline (PBS) at a concentration of 10 μg mL^−1^. 200 μL of the dextran solution was dispensed in each culture insert and 500 μL of PBS in the wells of a multiwell plate in which the culture inserts were mounted. At 30, 60, 90, 120, and 150 min, in each case, 50 μL samples of diffused dextran in PBS were aspirated from the bottom compartments. After each sampling, the liquid volumes in the bottom compartments were replenished with 50 μL of PBS to keep the volumes constant. The samples were analyzed by measuring their fluorescent intensity in a CLARIOstar plate reader (BMG LABTECH). The dextran amounts could then be determined using a calibration curve. Finally, the apparent permeability^41^ was determined. It is defined as *P*_app_ = (d*Q*/d*t*) × 1/(*A* × *C*_0_), where d*Q*/d*t* is the linear appearance rate of the dextran in the bottom compartment, *A* is the exposed membrane area, and *C*_0_ is the initial concentration of the dextran in the top or “donor” compartment—the culture insert. The determination was through the linear curve fit of the data points of the amount of accumulated dextran in the bottom or “receiver” compartment—the well beneath the insert—at the discrete time points. The permeability testing was done for three membranes of both types, the formed, curved and the unformed, flat one (*n* = 3).

### 2.2 Microfluidic Chip Construction, Fabrication of Chip Housings, Assembly of Housings and Microcurved Membranes, Preparation for Cell Culture, and Removal of Membranes from Chips

The 3D lung-on-chip device comprised a top and a bottom housing half made from PDMS containing microfluidic structures, and the microcurved PC membrane sandwiched in between (Figure 2B). This configuration resulted in two stacked cell culture chambers, a top and a bottom one. These could be perfused independently through dedicated microfluidic inlet and outlet channels and ports, with the chambers being separated by the permeable culture support.

The housing halves were fabricated by casting and thermal curing of the PDMS precursor^42^ (Figure 2C). For this purpose, first, a micromold was produced by photolithography in an SU-8 epoxy resist (NANO SU-8 100, Micro Resist Technology) on a 525 μm thin 4-inch-diameter silicon wafer (Si-Mat Silicon Materials). On a WS-650Mz-23NPPB spin processor (Laurell Technologies), an around 400 μm thick resist layer was coated in a single step on the wafer, followed by a soft bake on a HP61A-2 programmable hot plate (Torrey Pines Scientific). Through a high-resolution polyester film mask (Micro Lithography Services), the resist was exposed for 2 times 30 s in a UV-KUB 2 UV LED-based exposure and masking system (KLOÉ; monochromatic light source; wavelength: 365 nm; intensity/power: 25 mW cm^–2^; so with a total dose of 1500 mJ cm^–2^) followed by a post-exposure bake (same hot plate as before). The (exposed) resist was developed in PGMEA (propylene glycol monomethyl ether acetate; Sigma-Aldrich), rinsed in ultrapure isopropanol, blow-dried with a nitrogen gun, and finalized by a hard bake (same hot plate as before). Apart from two small isolated (support) structures, see Section 3.2, the design of this mold was the same for the top and the bottom housing half. Then, the PDMS base resin and curing agent (SYLGARD 184, Dow) were mixed in a 10:1 w/w ratio, briefly degassed in a vacuum desiccator, cast on the mold, and cured at 80 °C for 2 h. After that, the structured PDMS layer was peeled off. Prior to assembly, four microfluidic ports in the form of through-holes were punched into the top housing half, and two vias into the membrane. This made it possible to microfluidically access both the top and the bottom compartment through the top housing half.

To assemble, covalently bond, and seal the three different parts of the chip (Figure 2D), based on a similar protocol from Tang and Lee,^43^ the microcurved PC membrane and the PDMS housing halves were cleaned with 70% ethanol and exposed to oxygen plasma (at 200 W for 15 s; Diener). Then, the membrane and the housing halves were immersed in (3-glycidoxypropyl)trimethoxysilane (GPTMS; 98%; Aldrich) and a 5% aqueous solution of (3-aminopropyl)triethoxysilane (APTES; 97%; Aldrich), respectively, in each case at 50 °C for 1 h. Afterward, all parts were rinsed thoroughly with deionized (DI) water, blow-dried with nitrogen, and assembled. Thereby, the membrane was oriented with the openings and concave side of the microwells, with its front or “top side”, pointing upward toward the top compartment, and with the convex side of the microwells, with its back/rear or “bottom side”, pointing downward toward the bottom compartment. The assembly was softly clamped together at room temperature (RT) for 1 h, followed by a post-annealing bake at 80 °C for 2 h to increase the bond strength. A cross-section of the assembly was visually inspected using a Versa 3D SEM (FEI).

Prior to cell culture, the chip compartments were wetted and sterilized with an ethanol−water series at decreasing concentrations of ethanol and eventually washed with sterile DI water. The membrane was then functionalized with a 0.2% gelatin-based coating solution (PELOBiotech) by infusing the solution into the top compartment of the microfluidic chip and incubating it at RT for 12 min. In the case of the coculture, the membrane was additionally functionalized with a “Speed Coating Solution” (PELOBiotech) by infusing the solution into the bottom compartment of the microfluidic chip and incubating it at RT for 5 min. During this time interval, the device and with it the membrane was turned and kept upside down.

After cell culture, the membrane was removed from the chip for further processing and analysis of the cells on the membrane. For this, first, the chamber area was cut out from the chip using a scalpel, thereby cutting completely through the chip and in a square shape around the circular chamber. Then, the top and the bottom PDMS housing halves were peeled off from the PC membrane using tweezers.

### 2.3 Computational Fluid Dynamics Simulation

The medium flow through the top and bottom culture chamber of the microfluidic lung on chip was modeled in COMSOL Multiphysics 5.4. The computational fluid dynamics (CFD) simulation was run as a laminar and steady/stationary flow problem, and, due to the rather complex geometry of the culture chambers, in 3D. The boundary conditions at the compartment walls were set to “no slip”. The cell culture media perfusing the top and bottom chambers was approximated as a (incompressible) Newtonian fluid that concerning its physical/inertial and rheological properties is identical with water at 37 °C and the properties of which are constant over time. For a first rougher estimation of the flow field, this can be considered as being sufficiently accurate. Under culture conditions, differences in the density of both fluids are practically nonexistent and, depending on the medium and its supplements, for the (dynamic) viscosity differences are in the percent to low ten percent range.^44^ Also, as continuously fresh medium was pumped from a syringe through the chip into a waste reservoir (via tubing sections in between), the composition and consequently the flow characteristics of the medium in the chip chambers did not change over time. The volumetric flow rate was set to 25 μL h^–1^, see also Section 2.4, and the (gauge) pressure at the transition from the culture chamber to the outlet channel was set to 0 Pa. The calculated flow fields were translated into heat maps of the shear rate. The used solver was a stationary one, and segregated for pressure and velocity. The solver’s relative tolerance was set to 0.001. The mesh type used was “coarser mesh”, and the resulting number of elements was more than half a million (607 405).

### 2.4 Microfluidic 3D Cultures of Alveolar Epithelial Cells Submerged and at the Air−Liquid Interface

The ALI culture of alveolar epithelial cells is required for their (further) differentiation including polarization. For the ALI culture, commercially available primary human alveolar epithelial cells (HAECs; PELOBiotech/Cell Biologics) were used. Prior to culture on chip, the HAECs were expanded in cell culture flasks coated with a 0.2% gelatin-based solution (PELOBiotech) in “Complete Epithelial Cell Medium” (PELOBiotech). The kit of this epithelial growth/proliferation medium included basal medium, epithelial cell growth factor supplement, 5% fetal bovine serum (FBS), and 1% antibiotic−antimycotic solution. The medium was refreshed every 2–3 days.

An ALI culture needs to be preceded by a submerged culture. During this preculture, the cells could grow to confluence, which is a prerequisite for the subsequent ALI culture. For the submerged culture, the HAECs were seeded on the top side of the functionalized membrane at a density of around 50 000 cells cm^–2^ by infusion of a suspension of these cells in epithelial growth medium through the access holes into the top compartment of the microfluidic chip. The same epithelial growth medium but without cells was also infused into the bottom compartment. The cells were allowed to settle on and adhere to the culture membrane for 3 h. Then, both chip compartments were perfused with the epithelial growth medium using 6 mL syringes mounted in a multisyringe rack of a syringe pump (World Precision Instruments) and through tubes with an inner diameter of 500 μm and an outer diameter of 1.5 mm (Tygon, SynVivo), press-fitted into the four microfluidic ports of the chip. The chip was perfused at a lower volume flow rate of 10 μL h^–1^ overnight to avoid cell detachment. Next, the volume flow rate in both compartments was increased to 25 μL h^–1^. The volume of each of the two circular cylindrical culture chambers in the case of the hexagonal microwell array was around 20 μL. Against this background, the applied 25 μL h^–1^ volume flow rate corresponds to a complete exchange of the culture medium in the top or bottom chamber every 48 min. This was 60–90 times more often than in the case of changing media on top of a monolayer typically every 2–3 days in a multiwell plate. However, the filling level/liquid column in a well plate is roughly 20 times higher than the height/depth of the medium-filled top or bottom compartment (or 10 times taking both compartments together). Altogether, at 25 μL h^–1^, the rate of continuous medium exchange of the culture in the microfluidic chip was roughly 3–4.5 times higher than the discontinuous one in a multiwell plate. The cells were cultured submerged for 7 days.

For the ALI culture following the submerged preculture, the HAECs in the top compartment were exposed to (incubator) air after stopping the medium flow in this compartment and aspirating the medium. Immediately after, in the bottom compartment, which continued to be perfused with the same medium as during the submerged culture, the volume flow rate was increased to 60 μL h^–1^. The reason for this increase was to partly compensate for the discontinued medium provision in the medium-emptied top compartment in terms of nutrient supply and metabolic waste removal. The gas exchange could occur by diffusion through filter (pipette) tips (S1120-3710, Starlab; 10/20 μL, graduated) press-fitted in the two ports of the chip accessing its top compartment and through the permeable chip housing from PDMS. After the 7 days of submerged culture, the cells were cultured at the ALI for another 14 days, that is 21 days of culture in total. During the ALI culture, the cells were washed every 3–4 days with PBS to remove potentially secreted mucus. For the cells to settle and adhere and for the submerged and ALI cell culture, the chips were kept inside an incubator at a temperature of 37 °C and in a 5% carbon dioxide atmosphere.

### 2.5 Microfluidic 3D Coculture of Submerged Lung Epithelial and Endothelial Cells

For the coculture of lung epithelial and microvascular cells (Figure 1A), Calu-3 human bronchial adenocarcinoma cells (HTB-55, ATCC), a model cell line for lung epithelial cells, and primary human lung microvascular endothelial cells (HLMVECs; PELOBiotech) were used. For the justification of choosing the Calu-3 cell line, even if not being of alveolar origin, see Section 3.7. Calu-3 cells were expanded in cell culture flasks coated with a 0.2% gelatin-based solution (PELOBiotech) in Eagle’s minimum essential medium (EMEM; Lonza) supplemented with 10% FBS. HLMVECs were expanded in cell culture flasks coated with “Speed Coating Solution” (PELOBiotech) in microvascular endothelial cell growth medium (PELOBiotech). For both cell types, the culture medium was refreshed every 2–3 days.

For the submerged coculture, first, HLMVECs were seeded at a density of 50 000 cells cm^–2^ on the bottom side of the functionalized membrane. The cells were allowed to settle on and adhere to the culture membrane for 3 h. During this interval, the microfluidic chip was turned and kept upside down. Calu-3 cells were then seeded on the top side of the functionalized membrane, at a density of again 50 000 cells cm^–2^. The cells were allowed to settle and adhere again for 3 h. Finally, the coculture was perfused with a 1:1 mixture of EMEM and the endothelial growth medium in both compartments, supplied at a volume flow rate of 25 μL h^–1^. The cells were cocultured submerged for 11 days. For the cells to settle and adhere and for the cell culture, the chips were kept inside an incubator at a temperature of 37 °C and in a 5% carbon dioxide atmosphere.

### 2.6 Immunofluorescence Staining and Confocal Fluorescence Microscopy

The cells were fixed with 4% paraformaldehyde in PBS at RT for 30 min, washed with PBS, and permeabilized using 0.1% v/v Triton X-100 in PBS at RT for 10 min. Nonspecific binding sites were then blocked in CAS-Block (Thermo Fisher Scientific) at RT for 10 min.

Both for HAECs cultured (only) submerged and for HAECs cultured (later also) at the ALI, the cell nuclei and the F-actin were labeled with 4′,6-diamidino-2-phenylindole (DAPI; 1:70; Sigma-Aldrich) and phalloidin conjugated with Alexa Fluor 647 (1:100; Thermo Fisher Scientific), respectively. Both the submerged- and the ALI-cultured HAECs were also labeled with anticytokeratin 8 (CK8; 1:200; Abcam). For the HAECs cultured submerged, additionally, the tight junctions were labeled with antioccludin conjugated with Alexa Fluor 488 (1:500; Invitrogen). The submerged-cultured HAECs were also labeled with antivimentin (1:800; Dako). The ALI-cultured HAECs were additionally labeled with antiaquaporin 5 (1:200; Abcam) and antipro-surfactant protein C (pSPC; 1:250; Merck). For cocultured Calu-3 cells and HLMVECs, the cell nuclei of both cell types were labeled with DAPI (1:70; Sigma-Aldrich). Additionally, the Calu-3 cells were labeled with antioccludin conjugated with Alexa Fluor 488 (1:500; Invitrogen), and the HLMVECs with anti-CD31 (1:1000; Dako). For DAPI and phalloidin, the incubation temperature and time was RT and 30 min, and for all primary antibodies it was 4 °C and overnight, respectively.

The samples were mounted between microscopy slides and coverslips with ProLong Gold Antifade mountant (Invitrogen, Molecular Probes). The samples were imaged by confocal laser scanning fluorescence microscopy using a TCS SP8 STED (stimulated emission depletion) microscope (Leica Microsystems). The images were acquired as *z*-stacks with slices every 0.8–1.5 μm at a 63× magnification using an oil immersion objective (with a numerical aperture of 1.4).

### 2.7 Thickness Quantification of the Alveolar Epithelial Layer

The thickness of the monolayer formed by the HAECs on the microcurved membranes was quantified at day 7 of the submerged culture under flow. Samples were stained for nuclei, F-actin, and tight junctions as already described, see Section 2.6. The thickness of the alveolar epithelial layer was quantified in five different locations per each of two perpendicular cross-sectional images (re)constructed from the *z*-stack of confocal images per each of two to three microwells and per each of the culture membranes of three lung chips (*n* = 3) using Fiji.

### 2.8 Quantification of the Numbers of Cocultured Lung Epithelial and Microvascular Cells

Epithelial (Calu-3) cell and endothelial cell (HLMVEC) numbers were quantified for the coculture samples, which were based on the square microwell arrays. Cell nuclei and F-actin were stained with DAPI and phalloidin, respectively, as already described, see Section 2.6. The confocal images of a *z*-stack were in each case combined using the microscopy software to create a maximum intensity projection image. For the Calu-3 cells, the per culture membrane one to four acquired images and in total 11 images acquired at a 20× magnification in each case contained the area of four microwells including their margins extending to the boundaries of the adjacent microwell areas of the same size and square shape. Within each image, first, the mean area of the cell nucleus was determined by averaging the area of ten measured randomly chosen nuclei. Then, within the same image, the total area covered by (all) cell nuclei was determined. Finally, the cell number within the image was calculated by dividing the total area covered with cell nuclei by the area of the average cell nucleus. In this procedure, errors in the determination of both areas due to misalignment between the horizontal image projection plane and the sloping (curved) membrane surface on which the cells reside compensate each other. The quantifications were done for each of the culture membranes of four lung chips (*n* = 4) and in Fiji. The automatic quantification of the numbers of HLMVECs from projection images of the other sides of three membranes (*n* = 3) in Fiji basically followed the same procedure as described above for the Calu-3 cells. The application of this quantification procedure was necessary because for the Calu-3 cells it was partly challenging to clearly distinguish between individual cells.

### 2.9 Statistical Analysis

Each set of technical/material and biological/cell-covered membrane samples contained three or four independent replicates (that is, in the latter case, from three or four different chips) per experimental condition (*n* = 3/*n* = 4), except in two cases where there were two replicates (*n* = 2). The quantified data is presented as mean ± standard deviation. The microwell depth and pore diameter data was analyzed using a one-way ANOVA (analysis of variance) test in combination with a Tukey’s post-hoc test and the cell number data from the coculture, using a Student’s *t*-test. For the statistical significance levels, see the corresponding figure captions.

## 3 Results and Discussion

### 3.1 Biomimetic Membranes

**Figure 3:**
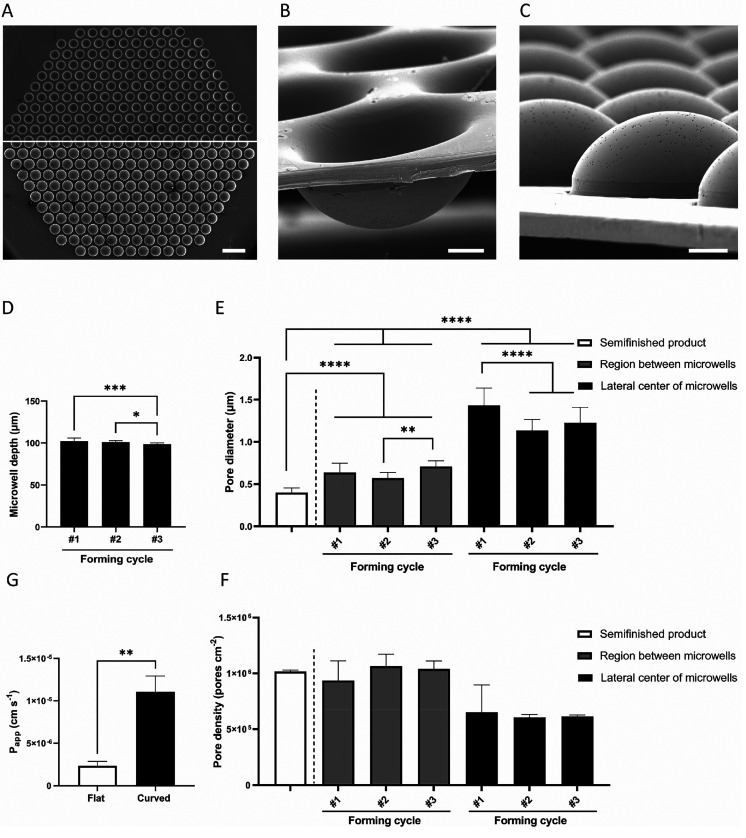
Visual inspection, geometrical characterization, and permeability testing of the biomimetically microcurved culture membrane from PC. (A) Top and bottom view of the microwell array (upper and lower image, respectively; SEM images; scale bar represents 500 μm). (B) Mixed top and side view of a section of the microwell array (SEM image; scale bar represents 50 μm). (C) Mixed bottom and side view of a section of the microwell array (SEM image; scale bar represents 50 μm). (D) Graph of microwell depths of formed membranes from three subsequent forming cycles (*n* = 3) (* and *** indicate *p*-values smaller than 0.05 and 0.001, respectively). Graph of (E) pore diameters and (F) densities of unformed membranes, and between and in the horizontal centers of the microwells of formed membranes from three subsequent forming cycles (*n* = 3) (** and **** indicate *p*-values smaller than 0.01 and 0.0001, respectively). (G) Graph of the apparent permeability of the unformed, flat membrane semifinished product and the formed, curved membrane (*n* = 3).

The cylindrical through-holes in the mold to form the biomimetically microcurved membranes were drilled with a diameter of 250 μm and a center-to-center distance between two adjacent holes of 300 μm, resulting in an interspacing of 50 μm (Figure S1). The mold for the hexagonal and square arrays comprised 3 × 3 = 9 and 2 × 4 = 8 arrays with in each case 325 and 81 through-holes, respectively. The microwells of the membranes were uniformly formed across the whole arrays (Figures 3A and S2), each with a maximum inner diameter at the concave−convex transition from the inner surface of the microwells to their circular upper rim of a little bit more than 200 μm (Figures 3B and S3A). The inner diameter was geometrically determined by the circular upper edges of the holes of the mold and by the slightly reduced membrane thickness at this location. The optimized parameters of the forming process, see Section 2.1, resulted in hemispherical microwells (Figures 3B,C and S3A,B) with an average maximum depth of 100.6 ± 3.0 μm (*n* = 3), which was very similar within and between different subsequent forming cycles (Figure 3D). The true depth of the microwells on the top side of the membrane is a little bit higher than the depth value derived from measurements on the bottom side of the membrane stated in the previous sentence (Figure S4). The microwells are deeper by the difference between the thickness of the membrane in the flat area between the microwells and the membrane thickness in the curved areas at their horizontal centers. The first thickness is nearly unchanged compared to the original and initial thickness of the membrane semifinished product of *t*_initial_ ≅ 25 μm before forming. The second thickness is reduced because of the stretching and consequently thinning down of the planar circular disc-type membrane area freely suspended over each circular-cylindrical mold cavity with an area *A*_circular disc_ into a microwell/–dome in the form of a membrane hemisphere with a curved area/surface *A*_hemisphere_ during membrane forming. When one assumes uniform membrane stretching, the reduced thickness can be estimated to be *t*_reduced_ = *t*_initial_ × *A*_circular disc_/*A*_hemisphere_ = *t*_initial_ × (π × *r*_circular disc/hemisphere_^2^)/(2 × π × *r*_circular disc/hemisphere_^2^) ≅ 25 μm × 1/2 = 12.5 μm. Thereby, *A*_circular disc_/*A*_hemisphere_ corresponds to the stretch ratio in this case.

Cross-sectional images of the microcurved membrane reveal cylindrical pores running perpendicular to the local membrane plane (Figure S5). In the flat area between the microwells and the curved areas at their horizontal centers, in both cases on the bottom side of the formed membranes, the average pore diameter and density of the unformed membranes of 0.40 ± 0.05 μm and (1.02 ± 0.01) × 10^6^ pores cm^–2^ (both *n* = 2) increased to an average diameter of 0.64 ± 0.10 and 1.26 ± 0.21 μm and decreased to an average density of (1.01 ± 0.11) × 10^6^ and (6.23 ± 0.55) × 10^5^ pores cm^–2^ (all *n* = 3), respectively (Figures 3E,F and S6A–C). The average pore diameter and density at the convex bottom side of the hemispherical microwell is a slight under- and overestimation, respectively. This is because of measurement errors due to the deviation of the imaging axis from the normal of the inclined, curved local film surface. The changes of the pore diameters and densities reflect the stretching of the membrane during forming. Thereby, the local pore diameter and density depends on the local stretch ratio, similar to the local pore length. The latter is practically identical with the local film thickness. These effects are partially compensating for each other in terms of their impact on membrane porosity and permeability. The apparent permeability of the formed, curved membrane of 11.06 × 10^–6^ cm s^–1^ is 4.6 times higher than that of the unformed, flat membrane of consequently 2.38 × 10^–6^ cm s^–1^ (Figure 3G). The above-mentioned pore diameters between 0.64 and 1.26 μm are in a wider range of pore sizes already successfully applied for membranes for the culture of lung epithelial cells over time. These values can be found in literature to be between 0.4 μm, for example, for PET membranes^45,46^ and 10 μm for PDMS membranes.^20^ In our membrane forming process, desired smaller or bigger pores in the formed end product are achieved with smaller or bigger pores in the unformed semifinished product. Ion track-etched membranes are available in a range of roughly 100 nm to 10 μm (and slightly above).

The process of pressure forming a film that is already porous before forming is in contrast to our so far conducted process in this respect. So far, the ion tracks of the heavy ion-irradiated film have been etched out to create the pores only after forming.^28,39,47^ The novel process on the one hand obviously additionally asks for a sealing film with suitable thermomechanical properties. At the forming temperature, the sealing film can already be a little bit softer than the porous film as it only has to seal the small pores, but on the larger scale of the mold cavities, it is supported by the porous film. On the other hand, the novel process saves one from the necessity of performing the final etching step in the case that simply commercial track-etched membranes are used, as demonstrated in this study. Despite a certain interlocking between the sealing film and the porous film after having been formed together, the separation of the first from the latter was easily possible without needing to apply too much pulling/peeling force (Video S1), which could damage the formed porous film and its microwells. The low separation forces were probably also because of the deformable RT mechanics and the nonstick properties of the material chosen for the sealing film. The peeling success rate for the hemispherical microwell structures with a maximum aspect ratio of consequently (around) 0.5 and a not high density of small pores was factually 100%. For this type of structure and the employed materials, similar success rates should be possible up to aspect ratios of 1–1.5.

Apart from our approach, there are some other processes for fabricating microstructured 3D porous culture membranes,^27^ such as by phase separation micromolding (PSμM),^48^ also including microscale curved features, such as by casting and curing of PDMS resin on top of curved and at the same time, on a smaller scale, pillared micromolds, the latter creating the columnar pores.^49^ For the fabrication of thin-walled 3D microstructures from porous membranes for cell culture, only a few approaches exist, such as the membrane micro emboss(ing) (MeME) process,^50^ which is also a process based on microthermoforming,^30^ and a process on the basis of collapsing or draping of porous membranes from partially cross-linked SU-8 over sacrificial silicon microstructures.^51^

While there is some early evidence of the effect of the curved concave surface of the microwells on alveolar epithelial cells, see Section 3.5, the cell-biologically beneficial effect of the obviously/visually more biomimetic design of the hexagonal over the square arrangement of the microwell array would still need to be proven. Of course, there are practically no differences in terms of the efforts for fabricating the arrays between the hexagonal arrangement and the square one both by the same micromolding process and using the same mold making technology. However, what can already be anticipated now is the benefit of the minimization of flat and convex surfaces—along with the cell populations residing on these surfaces—compared to concave surfaces for a number of nonimaging-based integrative biological readouts when considering the hexagonal instead of the square arrangement.^28^

### 3.2 Microfluidic Chips with Integrated Microcurved Membranes

**Figure 4:**
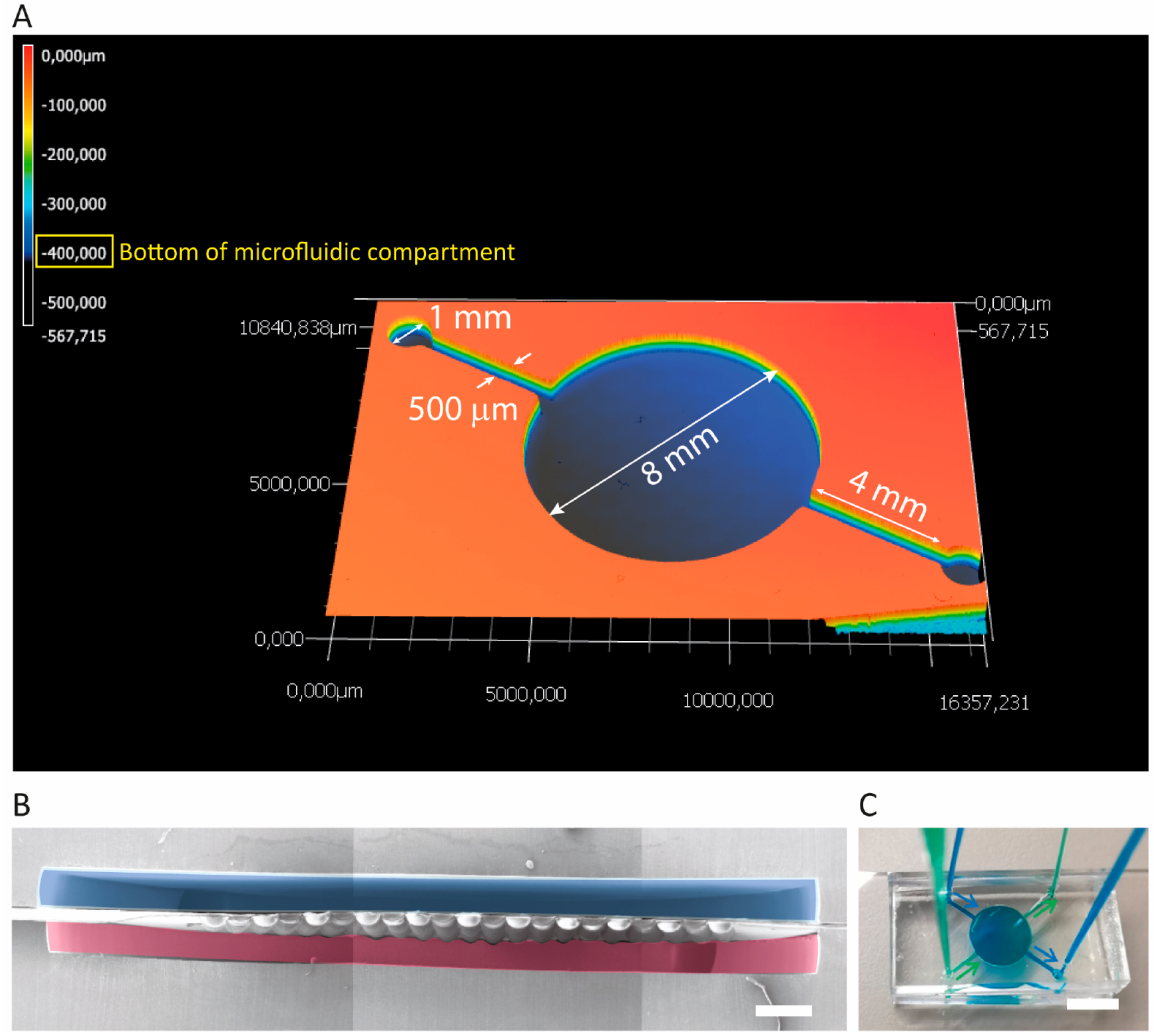
Geometrical characterization of the (bottom) housing half of the chip from PDMS, and perfusion and leak test of the assembled OoC device. (A) Each housing half of the microfluidic chip contained one of the two central circular culture chambers with a diameter of 8 mm for receiving the hexagonal microwell array. This chamber was on either side connected to an inlet and an outlet channel with in each case a width of 500 μm and a length of 4 mm. At their lateral/peripheral ends across the culture chamber, the two channels in turn were connected to in each case one smaller chamber with a diameter of 1 mm located in two opposite corners of the chip. The height/depth of the microfluidic compartments was around 400 μm. (B) Cross-section of an assembled 3D lung-on-chip device (stitched image; housing halves that during cell culture host epithelial and endothelial cells are colored blue and pink/purple, respectively; scale bar represents 500 μm). (C) Assembled lung-on-chip device with its top and bottom chip compartment perfused through press-fitted tubing with water colored with green and blue (food) dye, respectively (scale bar represents 8 mm).

Each housing half of the microfluidic chip contained one of the two central circular culture chambers with a diameter of 8 and 6 mm for the hexagonal and square microwell array, respectively (Figure 4A). This chamber was on either side connected to an inlet and an outlet channel with in each case a width of 500 μm and a length of 4 and 3 mm for the hexagonal and square array, respectively. At their lateral/peripheral ends across the culture chamber, the two channels in turn were connected to in each case one smaller chamber with a diameter of 1 mm located in two opposite corners of the chip. Additional, isolated 1 mm diameter chambers were placed in the two other (opposite) corners of the top housing half of the chip. The circular areas of these in total four smaller chambers also served as guiding/landing zones to support the manual punching of the microfluidic ports in the top housing half with a diameter of 500 μm. This was also the diameter of the two punched vias in the culture membrane. The intended height and depth of the microfluidic top and bottom compartments, respectively, was 400 μm, and the measured one was 402.2 ± 5.7 μm (*n* = 2), respectively (Figure 4A). The bonding based on the GPTMS and APTES functionalization of the PC membrane and the PDMS housing halves, respectively, resulted in an irreversible, blocking-free, and leak-tight assembly (Figure 4B,C).

### 3.3 Simulations

For each of the two culture chambers of the chip, the computed distribution of the shear rate is visualized in three horizontal sectional planes. In relative terms, in the top chamber, on the top side of the membrane, the shear rates at the (cell-populated) membrane surface are lower inside the shielded microwells and higher at the exposed hexagonal web between the microwells (Figures S7A–C and S8A–C); in the bottom chamber, at the bottom side of the membrane, the shear rates at the membrane surface are lower at the web between the microwells and higher at the horizontal centers of the microwells (Figures S7D–F and S8D–F). In absolute terms, even with the nutrient (and gas) supply situation being already more than sufficient, see Section 2.4, the shear rates are still very low. On the basis of a viscosity of the aqueous culture medium at 37 °C, which, as mentioned in Section 2.3, is (very) similar to that of water at the same temperature and around 6.92 × 10^–3^ dyn s cm^–2^, the maximum computed shear rates in the order of 0.1 s^–1^ can be converted into very mild shear stresses of 0.692 × 10^–3^ dyn cm^–2^. In another study by Huh et al. on a pulmonary edema (in a lung) on chip, after cell attachment, culture media was steadily flowed through both the upper/epithelial and lower/endothelial culture compartment at a volume flow rate of 50 μL h^–1^, exerting a fluid shear stress of around 0.2 dyn cm^–2^.^52^ This is roughly 2–3 orders of magnitude higher. The shear stress in capillaries *in vivo* was reported to be 10–20 dyn cm^–2^,^53^ which is again roughly 2 orders higher.

### 3.4 Formation of the Curved Alveolar Epithelial Layer On Chip

**Figure 5:**
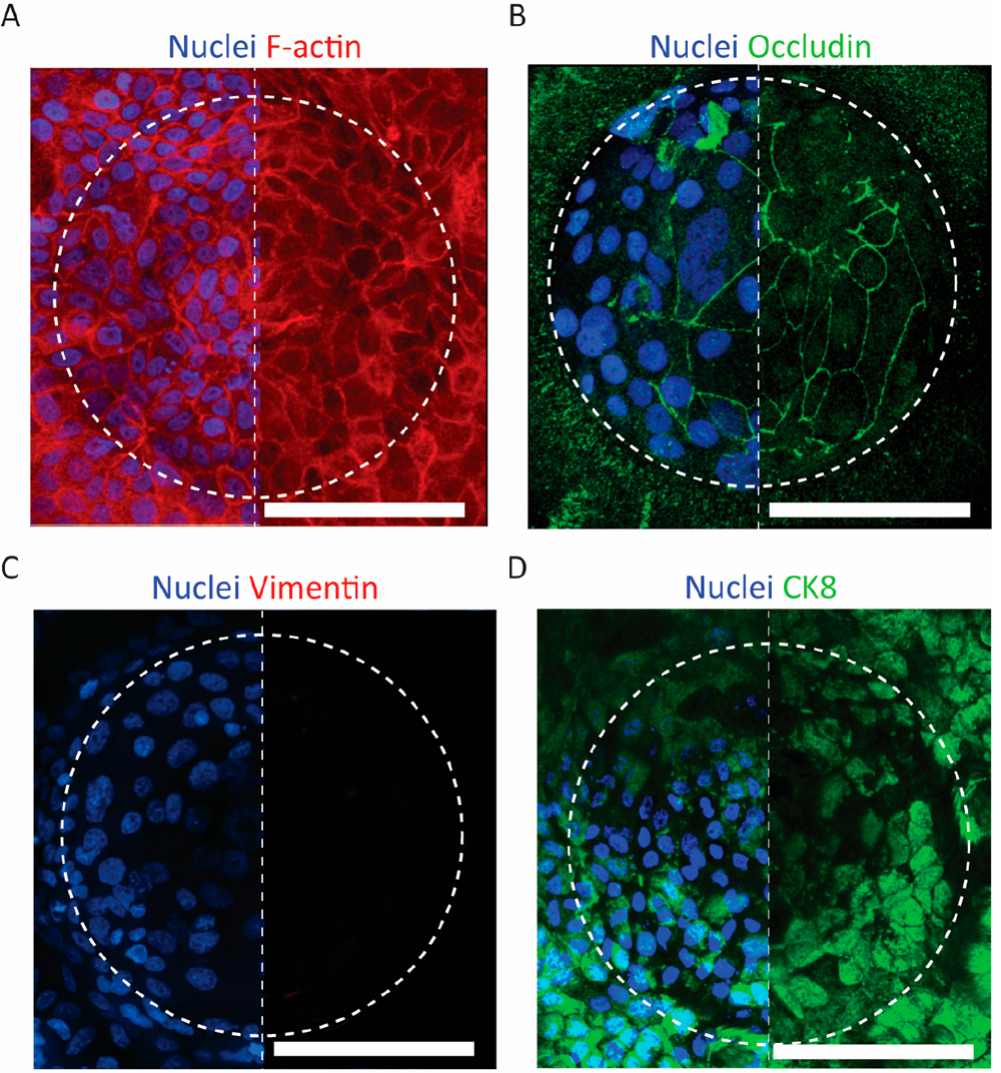
Epithelialization of the microcurved membrane in the chip. HAECs cultured submerged under flow for 7 days and stained for cell nuclei and (A) F-actin, (B) tight junctions, (C) vimentin, and (D) CK8 (fluorescent microscopy images; nuclei not shown in the right halves of the images for better visibility of the individual stains; scale bars represent 100 μm).

As already described, see Section 2.4, HAECs were infused into the top compartment of the chip, allowed to settle and adhere on the top side of the membrane, and cultured submerged under flow for 7 days. Subsequently switching to the ALI culture requires, as mentioned in Section 2.4, a confluent layer of the alveolar epithelial cells. Obviously, it was indeed possible to form confluent epithelial cell (mono)layers on the whole top side of the membrane (Figures 5A and S9A–C). These reveal the typical mesh-type patterns of tight junctions between the cells as evidenced by a corresponding expression of occludin (Figure 5B). An ALI-suitable coverage of this side of the membrane with cells was not necessarily to be expected. On the one hand, there was the pronounced topology of the membrane with the deepenings of the microwells and the elevations of the ridges in between. In gravity-seeding procedures, this typically leads to most cells landing at the deepest point of the wells. This bears the risk of an uncontrolled 3D aggregation/clumping rather than a defined 2D monolayer formation.^28^ On the other hand, there was the permanently sealed OoC device requiring seeding by infusion from the side of the culture chamber. This is in contrast to inoculation by dispensing from the top of the chamber, as it would be similarly possible in conjunction with culture inserts, for example. The infusion procedure normally leads to more cells upstream in the area of the chamber inlet and less cells downstream in the area of the chamber outlet. Despite these challenges, the cells fully lined the alveoli-like microwell structures of the membranes’ top side.

Due to an absent expression of vimentin at day 7 of the submerged culture, there was no indication that HAECs underwent epithelial-to-mesenchymal transition (EMT) (Figure 5C). At the same time, the cells were found widely positive for the epithelial marker CK8 (Figure 5D).

Interestingly, considerable differences in nucleus sizes and numbers and consequently cell sizes and numbers per microwell between the different microwells could be observed (Figure 5B,D). The cause of this inter-microwell variation is not yet known and understood, and obviously needs further investigation. One possible explanation for this phenomenon might be that it is the consolidated result of a variation of the seeding density. This in turn might be a consequence of the pronounced membrane topology discussed above.

### 3.5 ALI Culture of the Curved Alveolar Epithelial Layer On Chip

**Figure 6:**
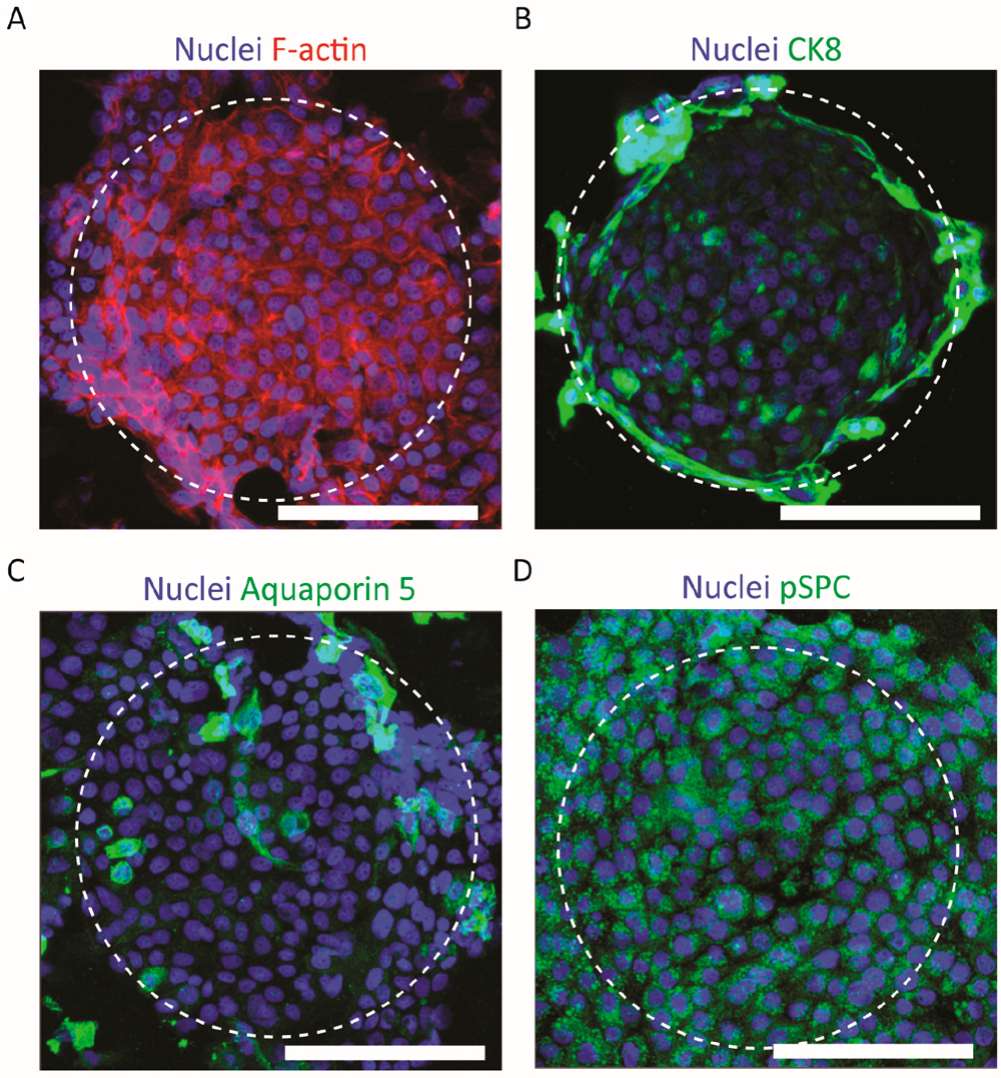
ALI culture on the microcurved membrane in the chip. HAECs cultured at the ALI under perfusion for 14 days and stained for cell nuclei and (A) F-actin, (B) CK8, (C) aquaporin 5, and (D) pSPC (fluorescent microscopy images; scale bars represent 100 μm).

The 3D lung-on-chip device was validated for its capability to sustain an ALI culture. For this, after an initial submerged culture under flow for 7 days, the formed confluent alveolar epithelial layer was exposed to air on the cells’ apical side while continuing perfusion with medium on their basal side (at an increased flow rate) for another 14 days. No leakage or inflow of medium from the bottom compartment through the cell-populated microcurved membrane into the top compartment was observed. This means that the epithelial cells remained exposed to air. After the 14 days of ALI culture, the microwells remained fully lined by the epithelial layer (Figure 6A). Similar to the 7 days of submerged culture, the HAECs kept expressing the epithelial marker CK8 also after the additional 14 days of culture at the ALI (Figure 6B). Furthermore, the HAEC layer revealed the expression of aquaporin 5, a marker of alveolar (epithelial) type (AT)I cells, only in a few spots (Figure 6C), but of pSPC, a marker of ATII cells, area-wide (Figure 6D). In mammalian lungs, the ATII cells are mainly located in the corners where adjacent alveoli meet.^54,55^ To some extent, a comparable distribution of pSPC expression over the alveoli-like microwell structures, which could have been an indication for a corresponding pattern of ATII cells, could unfortunately not be identified.

Occasionally, on the membrane removed from the chip, it could be observed, obviously as a consequence of cell detachment, that cells were partly missing on the ridges between the microwells. As already discussed, the cells in this area were relatively more exposed to flow than those in the concave inner of the microwells, see Section 3.3. However, as also discussed there, the shear rates were absolutely very low. Possibly, the detachment of cells resulted from the necessarily destructive removal of the membrane from the chip. Likely, however, it resulted from the relative movement of the microscope coverslip and the cell-covered membrane during mounting of the membrane with mounting medium as preparation for confocal fluorescent microscopy.

In a previous study, under static conditions and mounted in culture inserts, we investigated a porous membrane-based array of hemispherical microwells also in combination with growing, among others, HAECs on it.^28^ The membrane was not yet based on the biomimetic hexagonal arrangement of the alveoli-like structures and still created by a more laborious process for the introduction of the pores, see also Section 3.1. In the study, we observed distinct cellular responses to the membrane curvature. Cells on the curved membrane revealed significant differences compared to cells on a flat counterpart regarding membrane epithelialization, areal cell density of the formed epithelial layers, their cross-sectional morphology, and their proliferation and apoptosis rates, and the same tight barrier function as on the flat substrate.

### 3.6 Thickness of the Formed, Curved Alveolar Epithelial Layer

**Figure 7:**
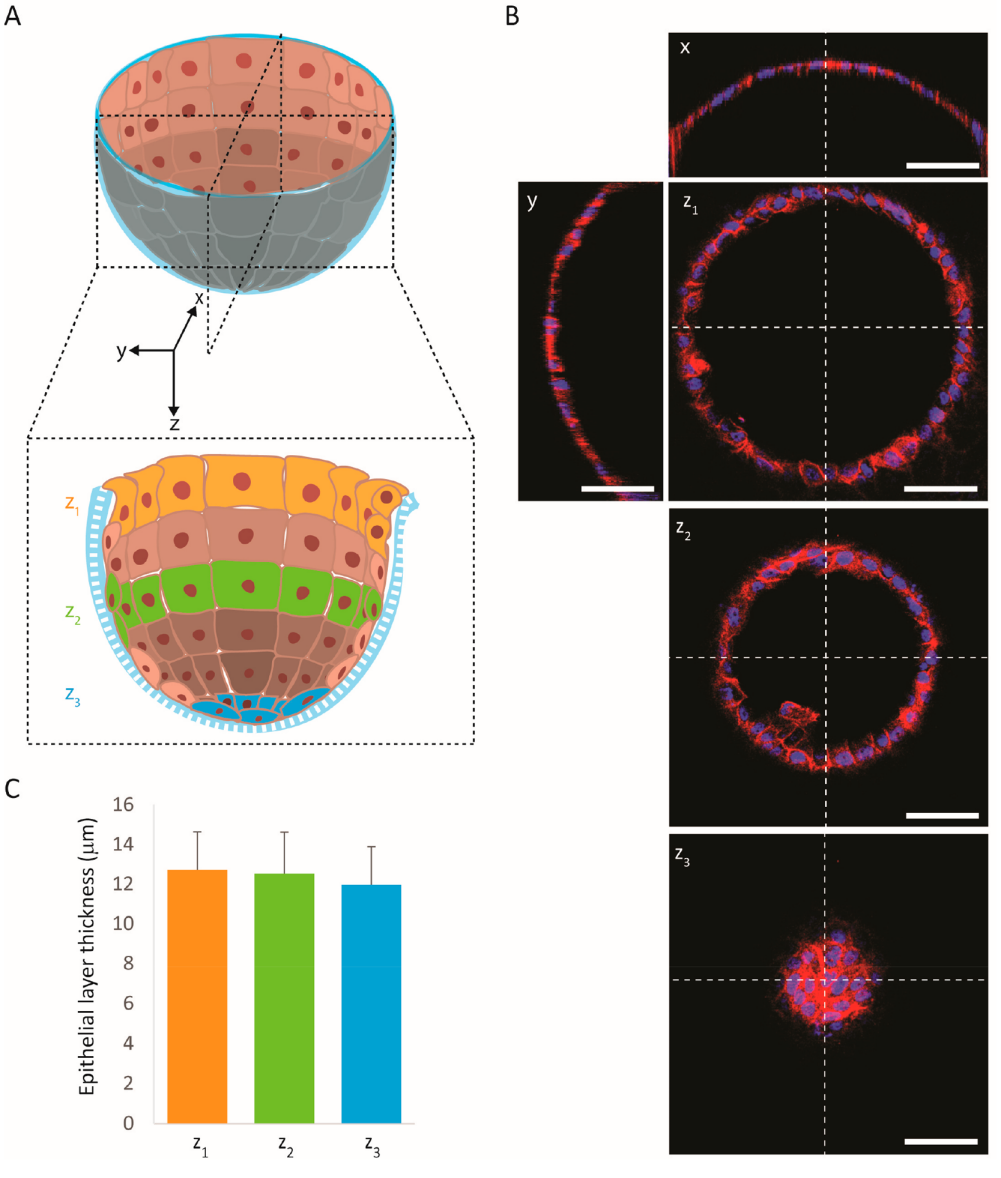
Thickness of the formed, curved alveolar epithelial layer. The thickness of the HAEC lining was measured (A) in two perpendicular cross-sections and there in each case in five different locations: at the horizontal center of the bottom of the microwell, at the left and right sidewall of the microwell directly under its convex rim, and at the left and right sidewall roughly halfway between, in each case perpendicular to the microwell wall. (B) Representative vertical and horizontal cross-sectional images of the epithelial layer (image planes “*x*” and “*y*”, and “*z*_1_” to “*z*_3_”, respectively; scale bars represent 50 μm). (C) Graph of the HAEC layer thickness as a function of the measurement location as stated in (A) (*n* = 3).

As already reasoned, see Section 3.4, the achieved complete coverage of the membrane with HAECs following their seeding on the top side of the membrane by overflowing the same with the cell suspension was not necessarily to be expected. This was similarly true for the obtained homogeneity of the formed, curved layer of the alveolar epithelial cells. The measurements of the HAEC lining from the constructed cross-sectional images of the HAEC layer as well as the view on representative vertical and horizontal cross-sectional images revealed an epithelial monolayer that already at day 7 of the submerged culture under flow was homogeneous in terms of layer thickness (Figure 7A–C). This was the case both over the depth/height of the microwell and its circumference (Figure 7B). The epithelial thickness was seemingly/obviously also not negatively affected by the flow and was the same in the up- and downstream direction. The measured thickness was around 12 μm (Figure 7C). This is similar to values reported for the thickness of the nuclear region of lung epithelial cells.^32^

### 3.7 Coculture of Lung Epithelial and Endothelial Cells On Chip

The 3D lung-on-chip device was also validated for its capacity to sustain a coculture of lung epithelial and microvascular cells. These two cell types are the key players of the alveolar−capillary barrier in the lungs. For the lung epithelial cells, Calu-3 cells were chosen. Even if not being of alveolar origin and if the curvature types and diameters experienced by epithelial cells in the large airways are different from those of the alveoli, Calu-3 cells were chosen as they are one of the few respiratory cell lines that form tight junctions *in vitro*.^34^ For the same reason, Calu-3 cells have been widely used in airway epithelial barrier studies.^35^ This is in contrast to A549 human lung adenocarcinoma epithelial cells, a model cell line for alveolar-type (AT)II cells. For A549 cells, there is a reported lack of formation of functional tight junctions when grown in (mono)layers.^37,56^ It is important to note that apart from circular hemispherical microwells the novel process variant also allows the forming of membrane microwells with all kinds of shapes. This can be straight or branched elongated hemispherical shapes mimicking key aspects of the anatomy of human airways, such as the terminal bronchioles, in terms of their average shape and size (Figure S10A,B).

**Figure 8:**
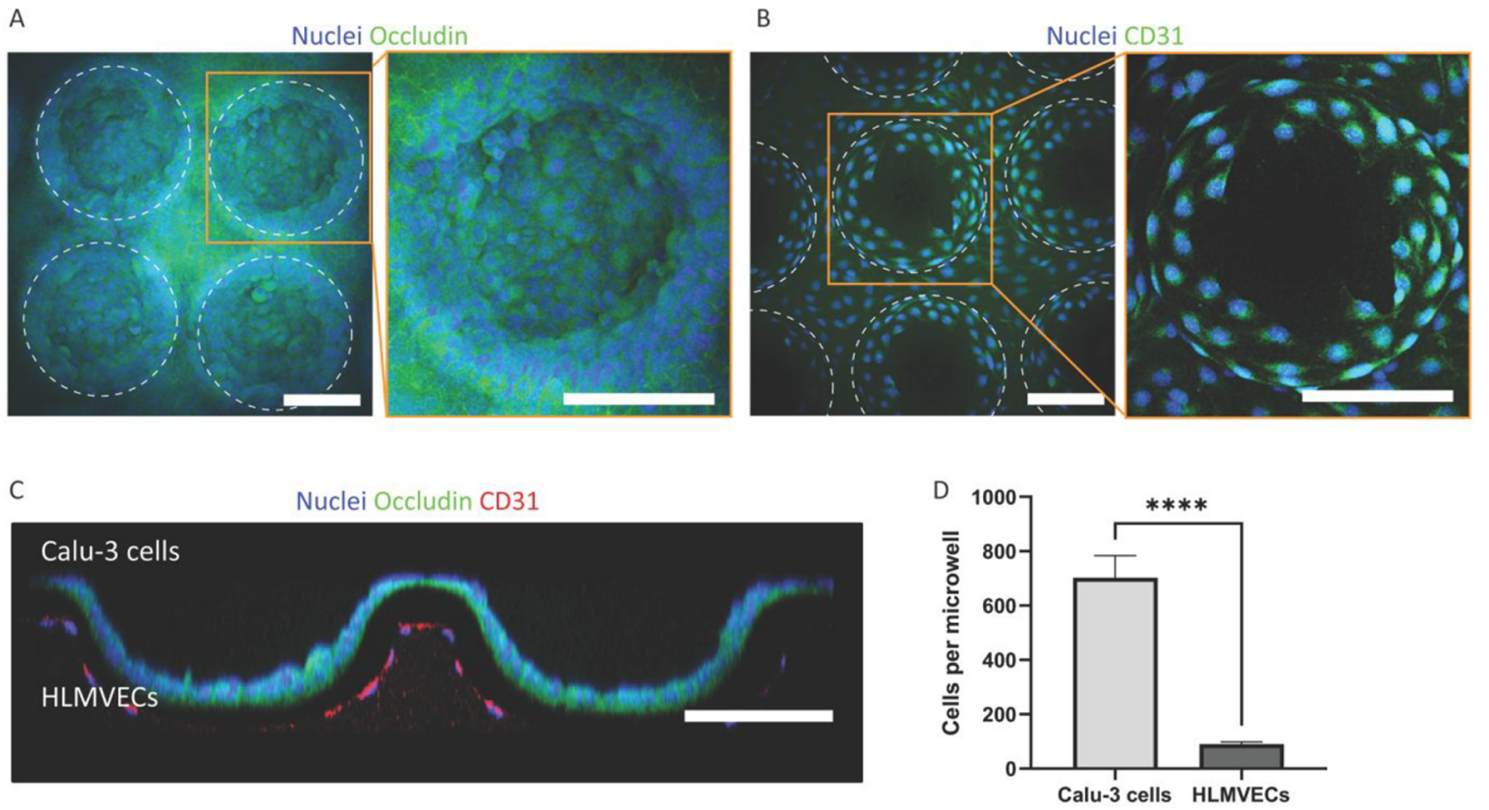
Lung epithelial and endothelial coculture on the microcurved membrane in the chip. (A) Top views of sections of the microcurved membrane with Calu-3 cells cultured for 11 days and stained for cell nuclei and tight junctions (fluorescent microscopy image; scale bars represent 100 μm). (B) Bottom views of sections of the same microcurved membrane with HLMVECs cultured for 11 days and stained for nuclei and CD31 (fluorescent microscopy image; scale bars represent 100 μm). (C) Cross-section of the microcurved membrane from (A) and (B) (scale bar represents 100 μm). (D) Graph of the count of Calu-3 cells (*n* = 4) and HLMVECs per square microwell unit (*n* = 3) (**** indicates a *p*-value smaller than 0.0001).

For the coculture, on either side of the membrane, HLMVECs and Calu-3 cells were seeded at the same density, allowed the same time interval to settle and adhere, and cultured in the same mixed medium under the same flow rate and for the same period of time. Similarly to the HAECs, also the Calu-3 cells formed confluent epithelial layers with mesh-type patterns of tight junctions between the cells on the whole top side of the membrane (Figure 8A). The epithelial cells completely covered both the concave and convex areas of this side of the membrane. Unlike the HAECs and Calu-3 cells, the HLMVECs did not completely do this on their side of the membrane (Figure 8B). They fully covered the flat area of the membrane’s bottom side and from the convex dome-type areas only the ring-type parts adjacent to the flat area. For both the Calu-3 cells and the HLMVECs, the cross-sectional view (Figure 8C) confirms the information on the spatial cellular distribution from the projection views (Figure 8A,B). The avoidance of the convex areas by the HLMVECs might in part be because of the fact that their luminal microenvironment is largely concave. The beneficial effect of this only partial coverage was a regular biomimetic cell pattern where the endothelial cells lined the interalveolar septum-like interspace between the microwells in a network-type fashion, as occurs in the natural counterpart. The quantification of the numbers of Calu-3 cells and HLMVECs, as mentioned above seeded with the identical density and then further handled and treated equally, revealed 705 ± 254 epithelial and 91 ± 7 endothelial cells per square microwell unit (Figure 8D). This corresponds to a ratio of around 7.7:1 of the two cell types.

## 4 Conclusions and Outlook

Here, we propose a more realistic and still robust culture environment for alveolar cells based on biomimetically microcurved track-etched membranes. In this feasibility study, the membranes were shaped into hexagonally arrayed hemispherical microwells by an innovative combination of 3D microfilm forming and ion track technology. The novel combined process presented itself as an alternative to our existing process combination with a reverse sequence of film forming and ion track/pore etching. A more thorough comparison between the two processes is beyond the scope of this study but should be part of a follow-up study. The 3D shaping of the culture membranes restored the mainly spherical geometry of the cells’ original microenvironment. Integrated in microfluidic chips where they separated a top from a bottom cell culture chamber, the microcurved membranes were seeded by infusion with primary HAECs. Despite the pronounced topology, the cells fully lined the alveoli-like microwell structures on the membranes’ top side. The confluent curved epithelial cell monolayers enabled the culture at the ALI for 14 days. Similarly, the top and bottom sides of the microcurved membranes were seeded with Calu-3 cells and HLMVECs, respectively. When doing so, the latter lined the interalveolar septum-like interspace between the microwells in a network-type fashion, as in the natural model. The coculture was maintained for 11 days.

The anticipated next steps toward even more realistic microenvironments for alveolar cells include, in addition to the hexagonal arrangement of the hemispherical microwells, a hexagonal design of their upper rim. This should then continuously blend into a spherical shape toward their bottom. This would even more approximate the topological landscape of alveolar tissue. It was already shown by us in the framework of another study in conjunction with topographically defined artificial cell microenvironments that it is in principle possible to generate such blended hexagonal-spherical microwell designs.^57^ The edges of the hexagonal mold cavities, however, require the one-time usage of an advanced micromold making process. Suitable process candidates are high aspect ratio-capable microlithography followed by electroplating/galvanoforming, or laser micromachining using a femtosecond pulsed laser. Further next steps also include (alveolar) lung tissue-like, flexible/elastic materials, such as polytrimethylene carbonate (PTMC),^58−60^ instead of the stiff PC membranes for the fabrication of the microwells. Together with a (chemically inert) grid-type support with a design similar to the mold for forming the microwell arrays, a controlled breathing-like periodic inflation of each individual microwell of an array mounted and suspended in the support could then be achieved. This inflation would then include a corresponding extension/dilation of the cell layer adhering to the concave microwell surfaces. The next consequent step in terms of biological realism is the substitution of the Calu-3 cells in their coculture with the HLMVECs for the HAECs and to combine this primary cell coculture with the ALI culture in the epithelial compartment. In such a setup, it could then also be investigated if the biomimetically patterned endothelial cell population benefits the epithelial population, as this could be already similarly shown for (nonpatterned) static Calu-3 cell-LMVEC-cocultures in culture inserts.^60^ The presented 3D lung-on-chip model might set the stage for (micro)anatomically inspired membrane-based OoC models of other epithelial and/or endothelial tissue barriers, such as of the bronchioles, renal tubules, intestinal villi, or blood or lymph vessels, in the future.

## Supporting Information Available

Microthermoforming mold from brass; porous PC microwell array; single porous PC microwell; optical profiler measurement of the microwell depth; perpendicularly oriented micropores in the formed PC membrane; differently sized and differently densely arranged micropores in an unformed and a formed PC membrane; CFD simulation for proofing of (critical) shear rates in the culture chambers of the chip; confluent epithelial cell monolayer on a microcurved membrane; examples of shapes that can be realized with the novel microthermoforming process variant (PDF)
Video S1: Separation of the formed porous film from the similarly formed sealing film by peeling them from each other (MP4)

## Abbreviations

3D: three-dimensional
APTES: (3-aminopropyl)triethoxysilane
ALI: air–liquid interface
AT(I/II): alveolar type (I/II)
CFD: computational fluid dynamics
CK8: cytokeratin 8
OPD: chronic obstructive pulmonary disease
DAPI: 4′,6-diamidino-2-phenylindole
DI: deionized
EMEM: Eagle’s minimum essential medium
EMT: epithelial-(to-)mesenchymal transition
FBS: fetal bovine serum
GPTMS: (3-glycid(yl)oxypropyl)trimethoxysilane
HAEC: human alveolar epithelial cell
HLMVEC: human lung microvascular endothelial cell
MeME: membrane micro embossing
OoC: organ on a chip
PBS: phosphate-buffered saline
PC: polycarbonate
PDMS: polydimethylsiloxane
PET: polyethylene terephthalate
PP: polypropylene
pSPC: pro-surfactant protein C
PSμM: phase separation micromolding
PTMC: polytrimethylene carbonate
ROI: region of interest
RT: room temperature
SEM: scanning electron microscope
STED: stimulated emission depletion
