# Supplementary material for: 3D Lung-on-Chip Model Based on Biomimetically Microcurved Culture Membranes
Source: ACS Biomater Sci Eng. 2022 May 3;8(6):2684–99. doi: 10.1021/acsbiomaterials.1c01463 (PMC9198974; doi:10.1021/acsbiomaterials.1c01463)
Supplement: Supplementary file 1 — ab1c01463_si_001.pdf [file ab1c01463_si_001.pdf]

Supporting information

3D lung-on-chip model based on biomimetically microcurved culture membranes

Danielle Baptista[1], Liliana Moreira Teixeira[1,2], David Barata[1,3], Zeinab Tahmasebi Birgani[1], Jasia King[1], Sander van Riet[4], Thijs Pasman[5], André A. Poot[5], Dimitrios Stamatialis[5], Robbert J. Rottier[6], Pieter S. Hiemstra[4], Aurélie Carlier[1], Clemens van Blitterswijk[1], Pamela Habibović[1,†], Stefan Giselbrecht[1,†], and Roman Truckenmüller[1,†,*]

[1]MERLN Institute for Technology-Inspired Regenerative Medicine, Maastricht University, Universiteitssingel 40, 6229 ER Maastricht, The Netherlands

[2]Department of Developmental BioEngineering, Technical Medical Centre, University of Twente, Drienerlolaan 5, 7522 NB Enschede, The Netherlands

[3]Instituto de Medicina Molecular, Faculty of Medicine, University of Lisbon, Avenida Professor Egas Moniz, 1649-028 Lisbon, Portugal

[4]Department of Pulmonology, Leiden University Medical Center, Albinusdreef 2, 2333 ZA Leiden, The Netherlands

[5]Department of Biomaterials Science and Technology, Technical Medical Centre, University of Twente, Drienerlolaan 5, 7522 NB Enschede, The Netherlands

[6]Department of Pediatric Surgery/Cell Biology, Erasmus (University) Medical Center Rotterdam – Sophia Children's Hospital, Doctor Molewaterplein 40, 3015 GD Rotterdam, The Netherlands

†these authors equally contributed to this paper

*corresponding author; e-mail: r.truckenmuller@maastrichtuniversity.nl

9 pages, 10 figures

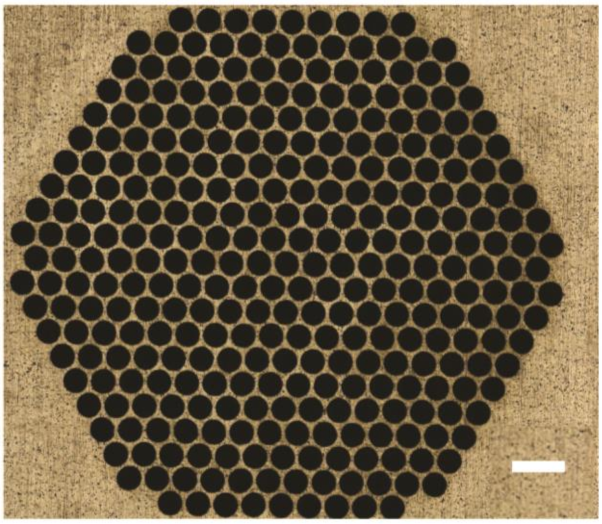

Figure S1. Microthermoforming mold from brass. The cavities of the micromold in the form of circular-cylindrical holes were fabricated by microdrilling (scale bar represents 500 µm).

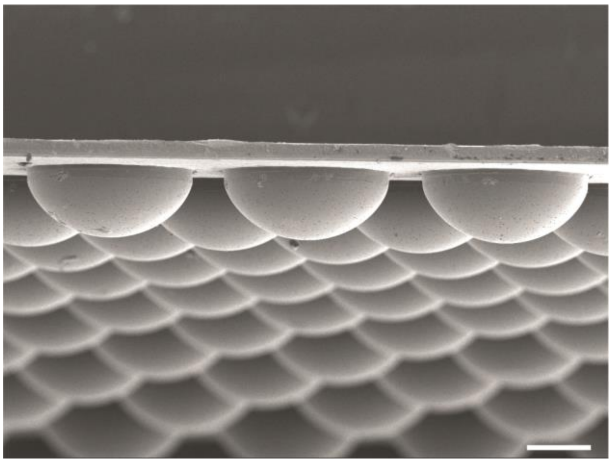

Figure S2. Porous PC microwell array. Mixed bottom and side view of a section of the array. The image reveals a uniform free-forming of the microwell array (SEM image; scale bar represents 100 µm).

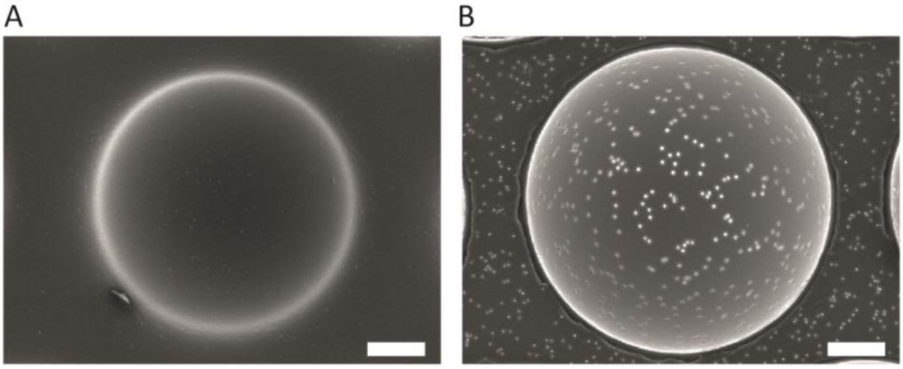

Figure S3. Single porous PC microwell. (A) Top and **(B)** bottom view. The image reveals a uniform free-forming of the hemispherical microwell (SEM images; scale bars represent 50 µm).

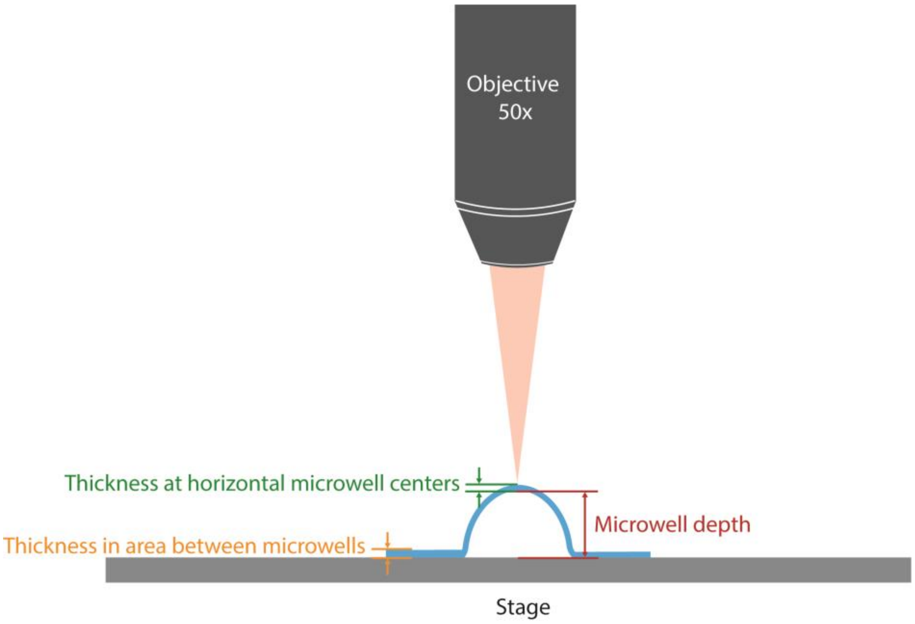

Figure S4. Optical profiler measurement of the microwell depth. For the determination of the depth of the microwells, they were measured on their backside. Then, to the measured (maximum) height difference, the difference between the thickness of the membrane between the microwells, which is roughly the original thickness of the unformed membrane semifinished product, and the thickness of the membrane at the bottom of the microwell, which is thinned down as a consequence of membrane stretching during the forming of the same, was added.

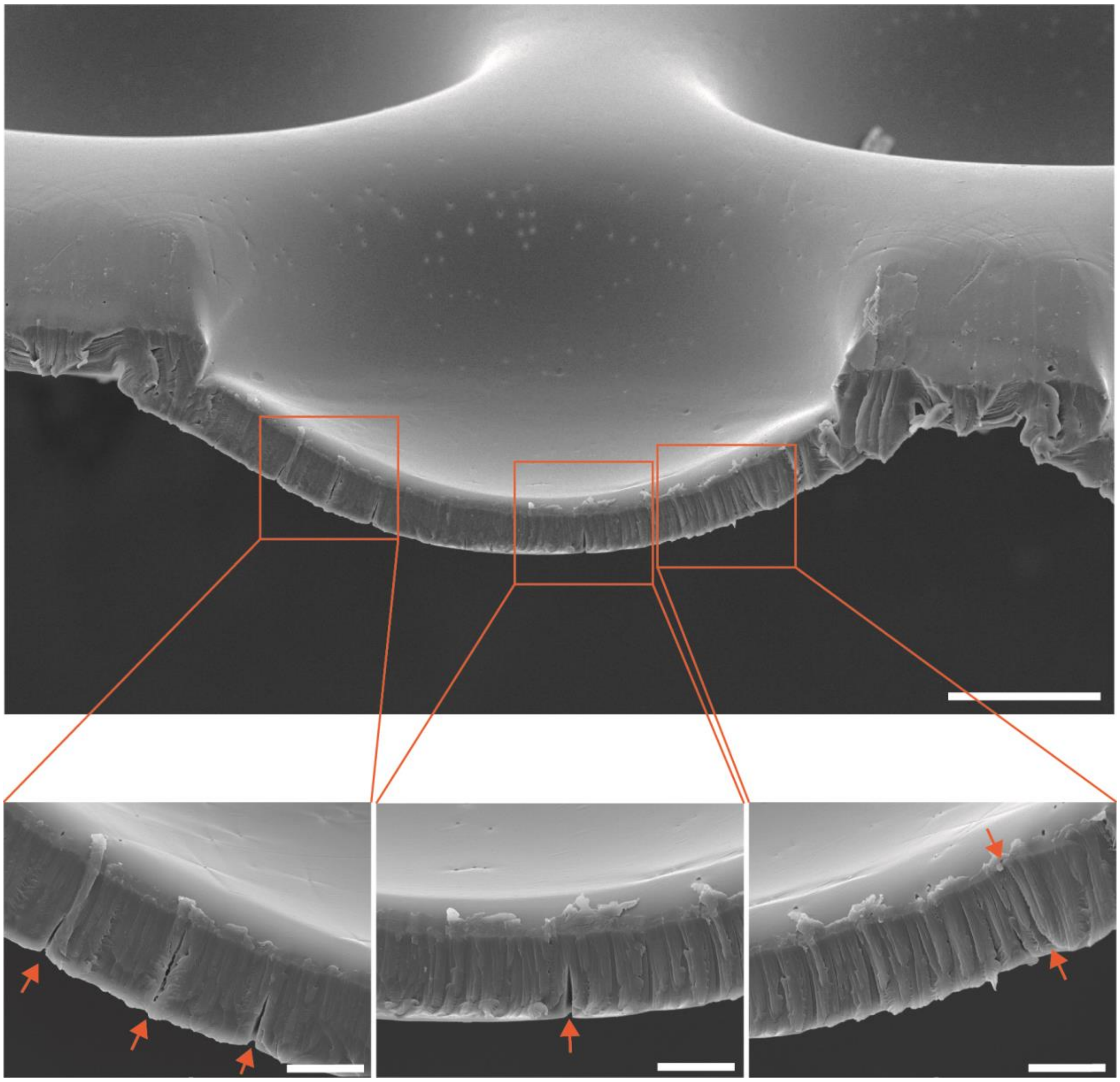

Figure S5. Perpendicularly oriented micropores in the formed PC membrane. The pores in the curved area(s) of a formed membrane are still perpendicularly oriented to the film plane as it was already the case in the unformed membrane semifinished product (bright red arrows indicate pores; the microwell is slightly flattened and the pore entries at the cross-section are slightly smeared up on the top side of the membrane because of cutting with a scalpel; SEM images; scale bars represent 50 and 10 µm for the top image and the zoomed-in bottom images, respectively).

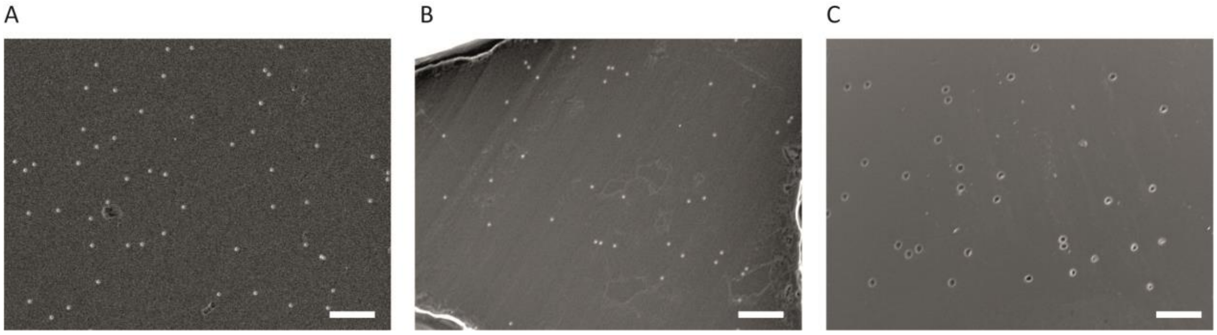

Figure S6. Differently sized and densely arranged micropores in an unformed and a formed PC membrane. Pores in **(A)** an unformed membrane semifinished product, and in **(B)** the flat area between the microwells and **(C)** a curved area at the horizontal centers of a microwell of a formed membrane on its bottom side (SEM images; scale bars represent 10 µm). As a consequence of stretching during forming of the membrane, the pores in the curved area(s) of the formed membrane are bigger/wider and less densely arranged than the ones in the flat area of the formed membrane and the unformed membrane.

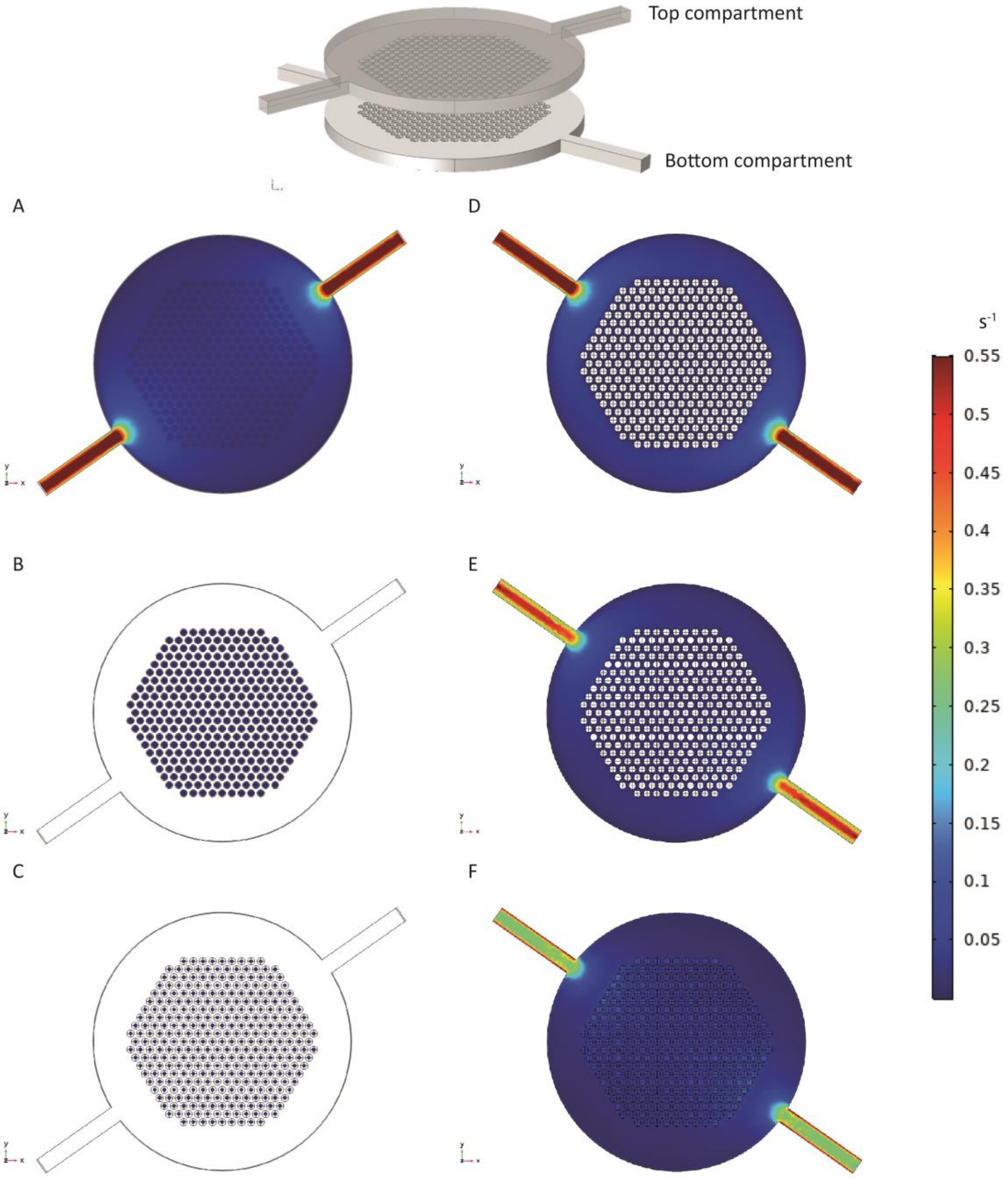

Figure S7. CFD simulation for proofing of (critical) shear rates in the culture chambers of the chip – part I. For each of the two culture chambers, the computed distribution of the shear rate is visualized in three horizontal sectional planes, and this over the full area of the chambers including parts of the inlet and outlet channels entering and exiting the chambers, respectively. For the top chamber, this is **(A)** directly above the hexagonal web between the microwells of the formed membrane, **(B)** at

half height in between the height level of (A) and (C; see next item), and **(C)** directly above the bottom of the microwells on the top side of the membrane. For the bottom chamber, this is **(D)** directly below the hexagonal web between the microwells, **(E)** at half height in between the height level of (D) and (F; see next item), and **(F)** directly below the microwells' bottom on the membrane's bottom side. The values displayed along the vertical color legend bar represent shear rates in [s^{-1}].

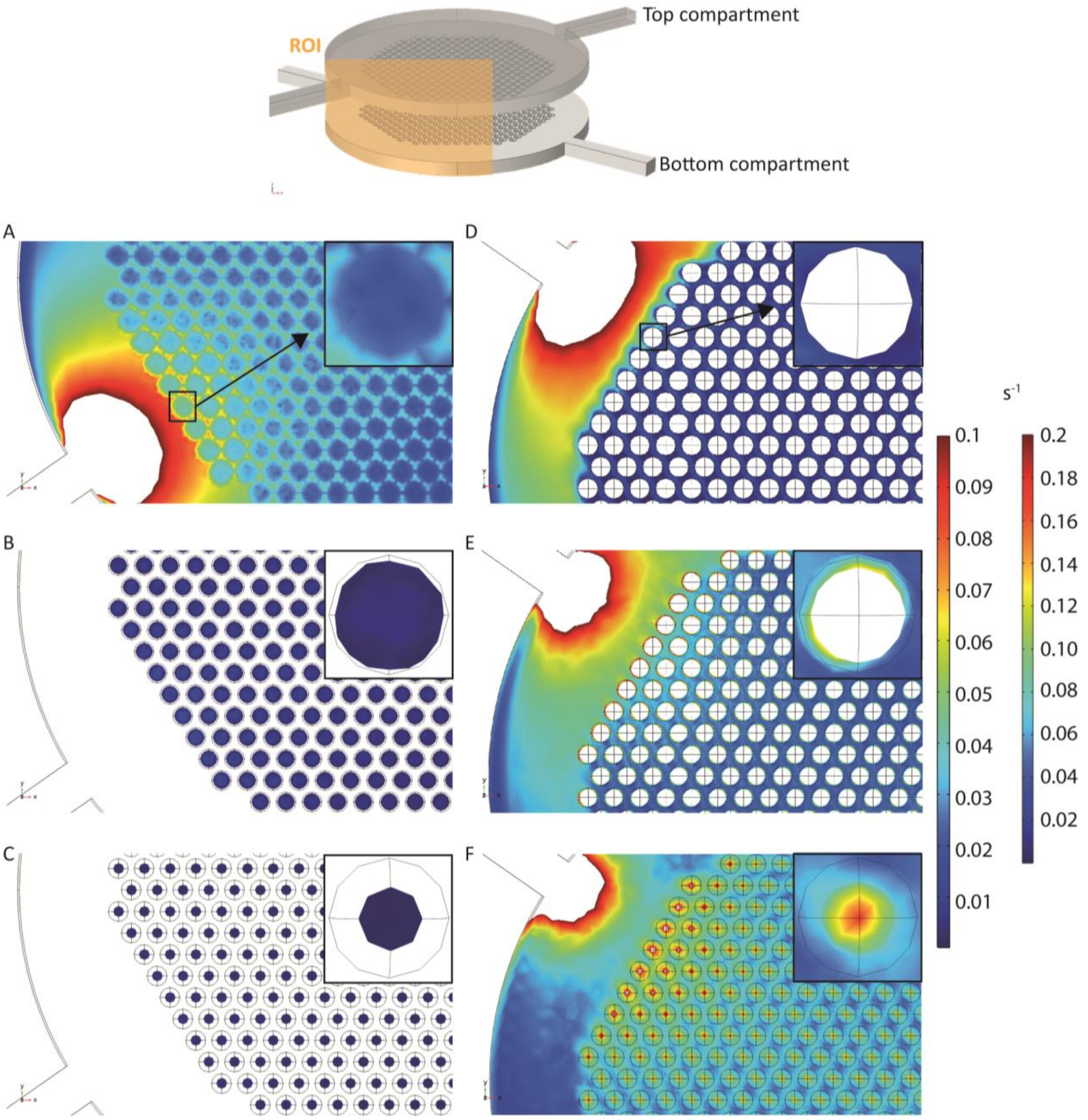

Figure S8. CFD simulation for proofing of (critical) shear rates in the culture chambers of the chip – part II. (A–F) Similar as for Figure S7A–F, for each of the two culture chambers, the computed

distribution of the shear rate is again visualized in three horizontal sectional planes. But now, the figure shows zoomed-in images from the regions where the top and bottom inlet channels enter the chambers, and further zoomed-in insets in the upper right corner of the main images from the regions around a single well as indicated in (A) and (D). The values displayed along the vertical color legend bars represent shear rates in [s^{-1}]; the left and right color legend bar relates to the main images and their insets, respectively. ROI: region of interest.

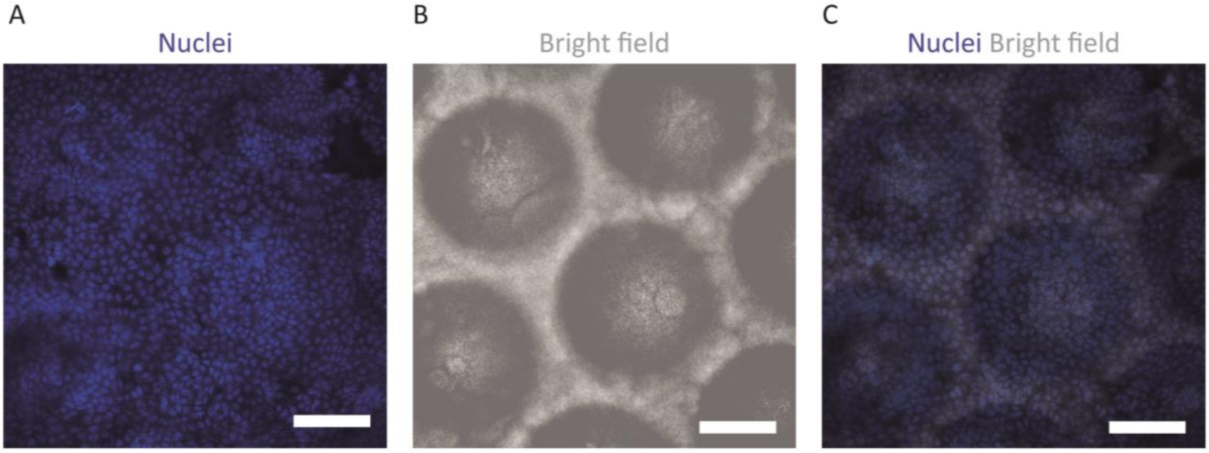

Figure S9. Confluent epithelial cell monolayer on a microcurved membrane. HAECs cultured submerged under flow for 7 days and stained for cell nuclei. Corresponding **(A)** fluorescent microscopy image, **(B)** bright-field microscopy image, showing the position of the microwells, and **(C)** combined image (scale bars represent 100 µm).

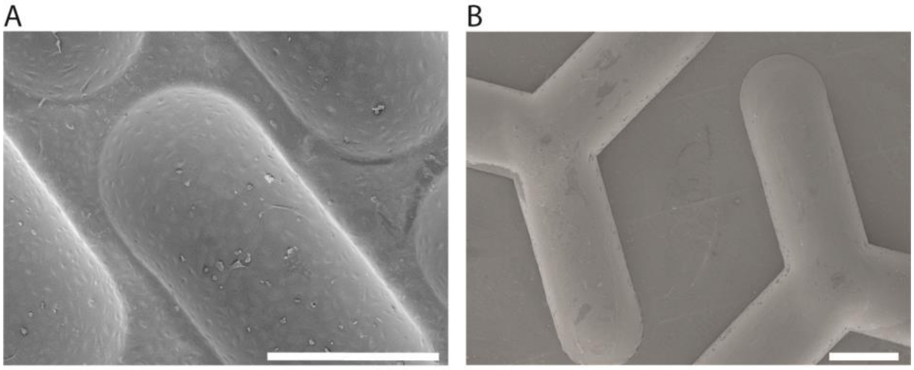

Figure S10. Examples of shapes that can be realized with the novel microthermoforming process variant. (A) Top view of a section of an array of straight elongated hemispherical microwells formed

in a 25 µm thin porous PC membrane (covered with bronchial epithelial cells; SEM image; scale bar represents 600 µm). **(B)** Bottom view of a section of an array of branched elongated hemispherical microwells formed in a 25 µm thin porous PC membrane (SEM image; scale bar represents 600 µm).
